# Electronic states of diarylethene switch–gold nanocluster hybrids

**DOI:** 10.1039/d6ra07292e

**Published:** 2026-10-30

**Authors:** Elham Mazarei, Evgenii Titov

**Affiliations:** a Universität Potsdam, Institut für Chemie Karl-Liebknecht-Str. 24-25 14476 Potsdam Germany mazarei@uni-potsdam.de titov@uni-potsdam.de

## Abstract

Diarylethenes (DAEs) are promising molecular photoswitches for optoelectronic devices and light-responsive nanomaterials. For potential applications, they can be mounted on metal surfaces or nanoparticles, which, in turn, may affect their switching behaviour. In this work, the electronic structure, reaction energetics, and optical properties of open- and closed-ring stiff DAE adsorbed on Au_*n*_ clusters (*n* = 10, 19, and 20) are investigated using density functional theory (DFT) and time-dependent density functional theory (TD-DFT). The latter is combined with fraction of transition density matrix (FTDM) analysis to characterize excited states by distinguishing between molecule-localized, gold-cluster-localized, and charge-transfer excitations. We find that interaction with gold clusters lowers the calculated ground-state isomerization barrier (through the formation of a molecular radical), reduces the energetic separation between the open and closed forms, and introduces new low-energy electronic transitions. The low-lying excited states of the hybrid systems show mixed molecular, cluster-localized, and bidirectional charge-transfer character. The obtained results demonstrate that nanoscale gold can tune both the energetics and the optical response of DAE photoswitches, providing microscopic insights essential for the design of light-controlled molecule–metal hybrid systems.

## Abstract 2

Gold–stiff DAE coupling introduces low-energy hybrid excited states with mixed molecular, metal-localized, and bidirectional charge-transfer character.

## Graphical abstract

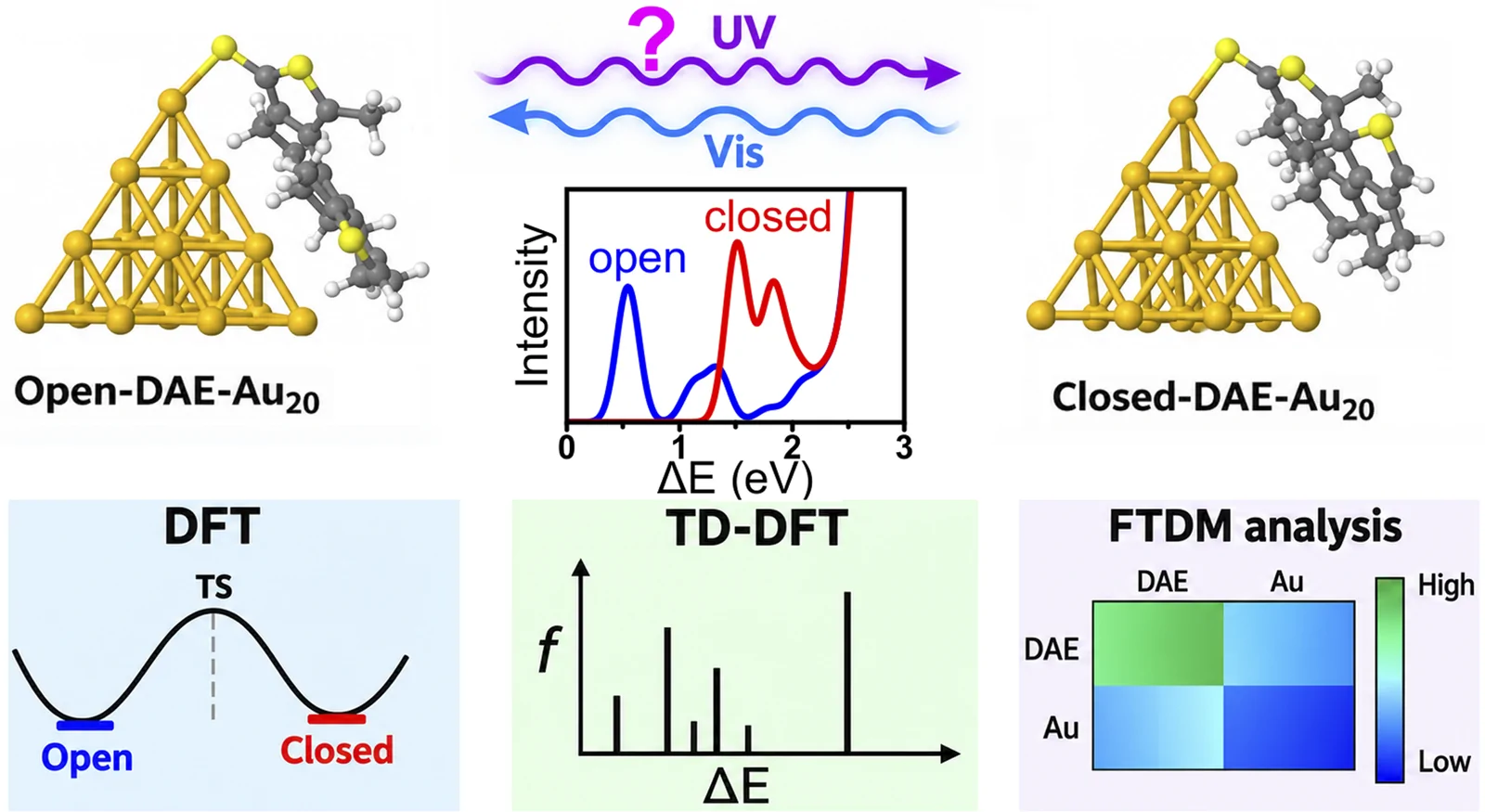

## 1 Introduction

To date, various types of photochromic molecules have been developed, including diarylethenes (DAEs), azobenzenes, spiropyrans, stilbenes, and fulgides.^1–4^ Among these systems, DAEs have attracted particular attention because they can reversibly switch between open-ring and closed-ring forms, which represent two metastable states, upon optical and/or electrical stimulation.^2,5^ DAEs are considered especially promising candidates for integration into thin-film devices because of their relatively small structural changes during isomerization, the thermal stability of both metastable states under ambient conditions, and their high fatigue resistance, even after repeated cycles of light irradiation.^5–9^ Owing to these properties, DAE-based molecular switches have been widely investigated for applications in optoelectronic devices,^1,9–12^ smart materials,^3,13^ and molecular machines,^14,15^ particularly when coupled to metal nanoparticles or metallic electrodes and surfaces.^16–20^

The switching mechanism of DAEs is based on a reversible ring-closing/ring-opening process, in which a carbon–carbon bond is formed or cleaved upon light irradiation.^5^ In many DAE systems, ultraviolet (UV) light induces the transition from the open-ring, less-conjugated and less conductive OFF state to the closed-ring, extended-π-conjugated and more conductive ON state.^2,5^ Conversely, visible light can promote the reverse transformation from the closed-ring to the open-ring form.^2,5^ However, one of the main challenges associated with DAE-based switches is the asymmetry of the photoisomerization process, as the ring-closing and ring-opening reactions often occur with different quantum yields.^5,21,22^ Structural modification of the DAE framework offers a strategy to tune this switching asymmetry.^23^ Highly constrained dithienylethenes (DTEs; thiophene-based DAEs) have been investigated as a way to control the conformational distribution of the open-ring isomer, because only the conformer with a suitable orientation of the reactive carbon atoms can undergo photoinduced ring closure.^23,24^

In these systems, alkyl chains of different lengths were introduced to covalently stabilize the photoactive conformation, leading to a more efficient formation of the closed-ring isomer upon UV irradiation, while the cycloreversion efficiency remained largely unaffected.^23^ In particular, a polycyclic dithienylethene, termed pcDTE_656_ in ref. 23 (see Fig. 1a), which locks the antiparallel conformation and fixes the central double bond, was designed.^23^ Here, the subscript “656” is part of the structural nomenclature of the polycyclic dithienylethene and refers to the ring-size pattern of the molecular framework.^23^ In the present work, we computationally study its complexes with gold nanoclusters.

**Fig. 1:**
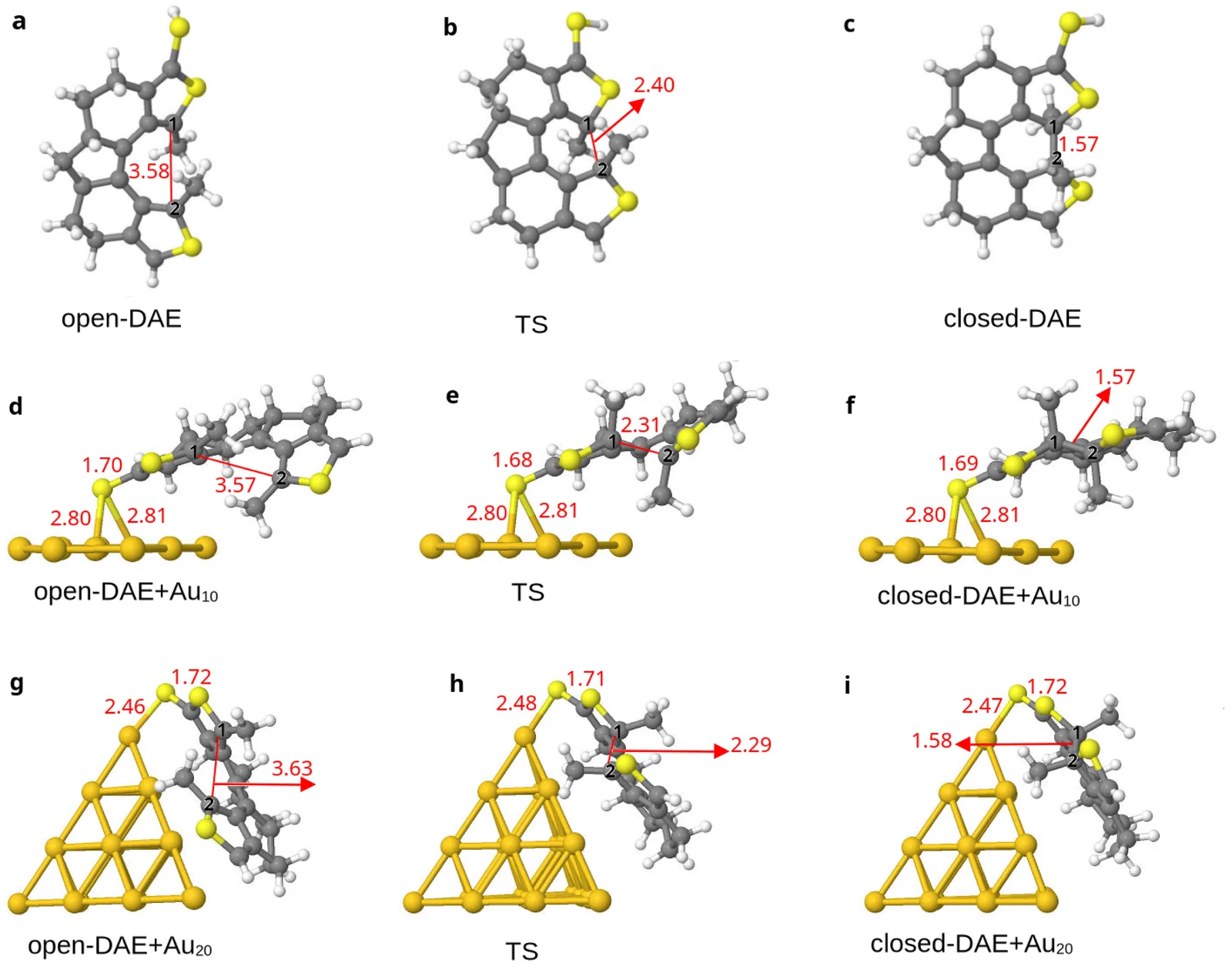
Optimized structures at the B3LYP-D3(BJ) level of theory along the switching reaction pathway of DAE. Panels (a) and (c) show the free open- and closed-DAE isomers, respectively, while panel (b) shows the TS structure connecting the open- and closed-forms. Panels (d–f) show the corresponding optimized structures of DAE adsorbed on the Au_10_ cluster, where the position of the linking S atom attached to Au was fixed. Panels (g–i) show the corresponding structures on the Au_20_ cluster, where the S atom was fully relaxed. Selected bond lengths are given in Å. The carbon atoms labeled 1 and 2 indicate the carbon centers involved in the ring-closing and ring-opening process. Dark gray, white, yellow, and golden-yellow spheres represent C, H, S, and Au atoms, respectively.

On the theory side, Perrier *et al.* performed a computational study (using density functional theory (DFT) and time-dependent DFT (TD-DFT)) on dithienylethene derivatives connected to small gold clusters (Au_3_ and Au_9_) and demonstrated that the interaction with gold can selectively affect the optical response of the two photochromic isomers.^25^ Their calculations showed that the closed-ring isomer largely preserves the electronic transition associated with ring opening, suggesting that the cycloreversion process can still occur after connection to gold clusters. In contrast, the absorption spectrum of the open-ring isomer is strongly perturbed by the gold cluster, which may suppress or hinder the ring-closing reaction.^25^ In a subsequent study, Perrier and co-workers further investigated the photochromic properties of dithienylethenes grafted on a larger Au_13_ cluster using a TD-DFT-based spectroscopic approach.^26^ By analyzing the electronic absorption spectra and the virtual molecular orbitals involved in the UV absorption bands, they showed that the attachment of the organic switch to a metallic cluster can either preserve or weaken its photochromic response. This work emphasized that the electronic coupling between the dithienylethene unit and the gold cluster plays a decisive role in determining whether the molecular switching function is retained after grafting onto metal nanostructures.^26^ Also, optical properties of complexes with even larger Au_19_ and Au_25_ clusters have been investigated using TD-DFT.^27^ Together, these studies highlight the importance of understanding metal–molecule interactions when designing DAE-based switches integrated with gold nanoparticles, electrodes, or plasmonic surfaces.

In this work, we investigate the complexes of the recently proposed DAE switch pcDTE_656_ (ref. 23) with gold nanoclusters (Au_10_, Au_19_, and Au_20_) using DFT and TD-DFT calculations. We focus on ground-state energetics and electronic absorption spectra, with specific emphasis on characterization of excited states using the fraction of transition density matrix (FTDM) analysis,^28,29^ allowing one to efficiently reveal molecular, metal, and charge-transfer contributions to the electronic transitions. The application of FTDM analysis and the investigation of the novel pcDTE_656_ switch represent new aspects compared with previous literature on DAE–gold systems.^25–27^

The Au_10_, Au_19_, and Au_20_ clusters are employed as finite nanocluster models to probe the local electronic coupling between the DAE molecule and nanoscale gold, rather than as models of an extended gold surface. We emphasize that the electronic properties of such small clusters can depend on their size and geometry and are not expected to be quantitatively converged with respect to a macroscopic gold support. Moreover, ligand passivation, solvent, and other environmental effects are not included in the present models. The results should therefore be interpreted primarily in terms of qualitative trends in molecule–gold coupling and its influence on the electronic and excited-state properties of the DAE switch.

Furthermore, the direct attachment of DAE to gold without an additional molecular spacer (see Fig. 1) is chosen deliberately to probe the regime of strong molecule–metal electronic coupling and to examine how this coupling affects the character of the low-lying excited states.

## 2 Computational details

### 2.1 Ground-state electronic structure calculations

The quantum chemical calculations in the present work were performed using the Gaussian 16 program package.^30^ Geometry optimizations and harmonic vibrational frequency calculations were carried out using the B3LYP exchange–correlation functional^31,32^ together with Grimme's D3 empirical dispersion correction with Becke–Johnson damping, denoted as B3LYP-D3(BJ).^33,34^ The 6-31G(d) basis set was used for the N, C, H, and S atoms,^35–37^ whereas the LANL2TZ(f) basis set/effective core potential was employed for the Au atoms^38^ (obtained from the Basis Set Exchange^39^). The structures were optimized for DAE adsorbed on Au_*n*_ clusters using two different approaches: in one case (Au_10_), the position of the S atom coordinated to Au was kept fixed, while in the other case (Au_20_), the molecular structure was fully relaxed without fixing the S atom. Gold atoms were always fixed.

The nature of each stationary point was confirmed by normal mode analysis (NMA). Local minima were identified by the absence of imaginary frequencies, while first-order saddle points (transition states, TSs) were characterized by the presence of a single imaginary frequency. In addition, we performed the intrinsic reaction coordinate (IRC) calculations^40,41^ to confirm that the located TSs indeed correspond to the isomerization reaction. The thermal isomerization rate constants were calculated using Eyring's transition state theory:^42^1
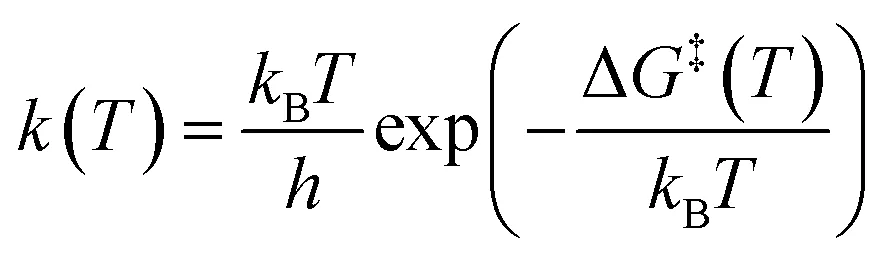
where *k*(*T*) is the thermal rate constant at temperature *T*, *k*_B_ is the Boltzmann constant, *h* is the Planck constant, and Δ*G*^‡^(*T*) is the activation Gibbs free energy at temperature *T*, defined as the Gibbs free energy difference between the transition state and the corresponding reactant minimum.

The vertical ionization potentials (VIP) and electron affinities (VEA) were calculated as

2a VIP = *E*_+_(0) − *E*_0_(0)

2b VEA = *E*_0_(0) − *E*_−_(0)

Here, *E*_+_, *E*_0_, *E*_−_ are the energies of the cation, neutral, and anion, respectively, and “(0)” denotes the optimized geometry of the neutral. The adiabatic ionization potentials (AIP) and electron affinities (AEA) were calculated as

3a AIP = *E*_+_(+) − *E*_0_(0)

3b AEA = *E*_0_(0) − *E*_−_(−)

where “(+)” and “(−)” stand for the optimized geometries of the cation and the anion, respectively.

The spin-restricted DFT (RDFT) was used for models with an even number of electrons (such as neutral free DAE (open- and closed-form) and open- and closed-form DAE adsorbed on Au_19_), whereas the spin-unrestricted DFT (UDFT) was used for the models with an odd number of electrons, namely cationic and anionic free DAE in the open and closed forms, as well as open- and closed-form DAE adsorbed on Au_10_ and Au_20_ clusters.

### 2.2 Calculation and analysis of absorption spectra

Vertical electronic absorption spectra were calculated for the pure gold clusters, the free DAE (open- and closed-form) molecules, and the gold-cluster–DAE hybrid systems in both the open- and closed-ring forms. The calculations were performed using time-dependent density functional theory (TD-DFT)^43^ at the B3LYP-D3(BJ) and ωB97X-D^44^ levels of theory. For several systems, test calculations were done using time-dependent Hartree–Fock (TD-HF) and configuration interaction singles (CIS). In all cases, the vertical excitation energies were calculated at the optimized B3LYP[-D3(BJ)] ground-state geometries. The stability analysis was performed prior to TD-DFT calculations using Gaussian's Stable(Opt) keyword, and the stable SCF (Kohn–Sham) solution was used in TD-DFT calculations. The nature of the excited states was analyzed using fraction of transition density matrix (FTDM)^28^ and natural transition orbital (NTO)^45^ approaches.

The vertical UV-Vis absorption spectra were simulated by convoluting the computed excitation energies and oscillator strengths with Gaussian functions according to:4
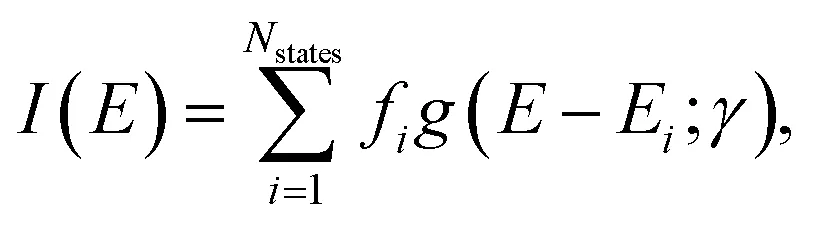
where *E*_*i*_ is the vertical excitation energy corresponding to the S_0_ → S_*i*_ transition, *f*_*i*_ is the associated oscillator strength, and *N*_states_ is the number of excited states included in the calculation (the first 50 excited states for B3LYP and 100 excited states for ωB97X-D). The Gaussian broadening function is given by:5
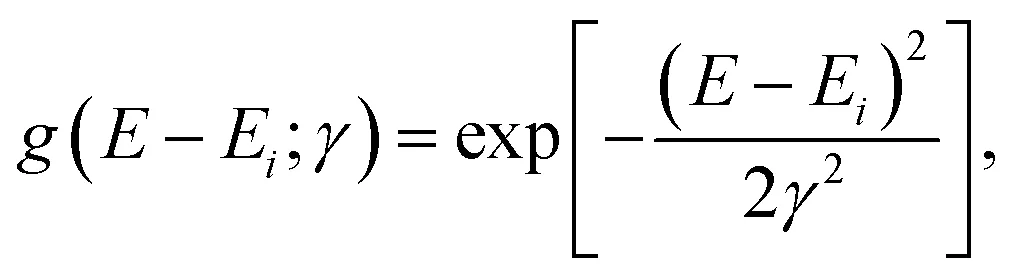
where *γ* is the width parameter (*γ* = 0.1 eV).

The FTDM analysis was used to quantify the localization and charge-transfer character of the excited states.^28,46,47^ For this purpose, the molecular systems were divided into chemically meaningful fragments, such as the DAE unit and the gold cluster. The fractional contribution of the transition density matrix from fragment *X* to fragment *Y*, for the transition from the ground state (0) to an excited state (*I*), was calculated as:^48,49^6
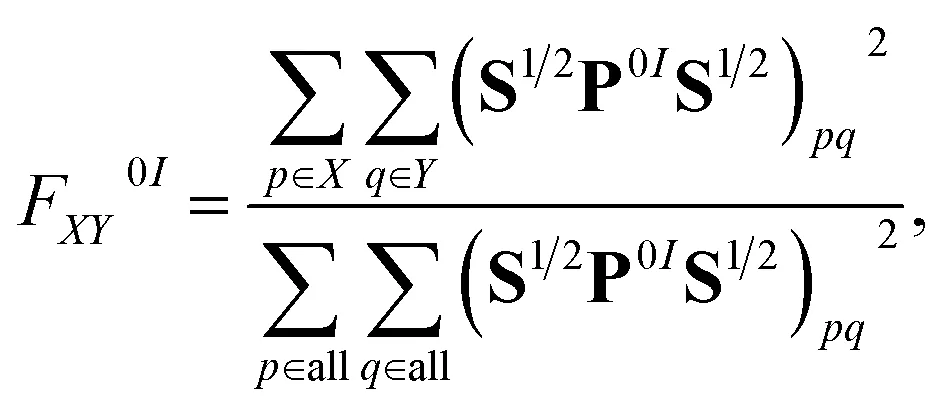
where **S** is the atomic-orbital overlap matrix and **P**^0*I*^ is the transition density matrix for the transition 0 → *I* in the atomic-orbital (AO) basis (computed with Multiwfn 3.8).^50,51^ In the case of spin-unrestricted calculations, this matrix is the sum of TDM matrices for α and β electrons. The sums run over AOs belonging to fragments *X* or *Y* in the numerator, and the entire system (“all”) in the denominator. We call the *F*_*XY*_^0*I*^ matrix the fraction-of-transition-density-matrix (FTDM) matrix. The diagonal elements *F*_*XX*_^0*I*^ represent local excitations (LE) (localized on fragment *X*), while the off-diagonal elements *F*_*XY*_^0*I*^, *Y* ≠ *X* correspond to charge transfer (CT) excitations (from fragment *X* to fragment *Y*). In the following, we will present the FTDM elements as percentages, *i.e.*, *F*_*XY*_^0*I*^ × 100%.

The resulting FTDM matrix elements provide a normalized measure of the transition density localized on, or transferred between, selected molecular fragments. Thus, this analysis allows the electron–hole localization and charge-transfer contributions of the excited states to be quantified, which is particularly useful for analyzing hybrid molecule–metal systems.^29^

In addition, to elucidate the character of the electronic transitions, NTO analysis was employed. NTOs provide a compact representation of the transition density matrix by expressing an electronic excitation in terms of dominant hole–particle pairs.^45,52,53^ This analysis facilitates the assignment of the main excitation character and allows one to distinguish between localized and charge-transfer transitions. It is used to complement the FTDM analysis for selected transitions.

## 3 Results and discussion

### 3.1 Ionization potentials, electron affinities, and absorption spectra of free open- and closed-form DAE

The structures of open- and closed-DAE are shown in Fig. 1a and c, respectively. The transition state (TS) of ring-closing/opening reaction is shown in Fig. 1b. The C_1_–C_2_ distance/bond length in free open- and closed-DAE is 3.58 and 1.57 Å at the B3LYP-D3(BJ) level of theory, respectively, and 2.40 Å for the TS.

Table 1 summarizes the vertical and adiabatic ionization potentials (VIP and AIP), and vertical and adiabatic electron affinities (VEA and AEA) of the open- and closed-form DAE. At the B3LYP level, the closed isomer exhibits lower VIP and AIP values than the open isomer, indicating that the closed form is more easily oxidized. In addition, the electron affinity changes from negative values for the open isomer to positive values for the closed isomer, suggesting that electron attachment is more favorable in the closed form. The vertical quantities obtained at the ωB97X-D level show the same general trend, supporting the conclusion that ring closure significantly modifies the redox properties of the DAE molecule.

**Table 1:** Vertical and adiabatic ionization potentials and electron affinities of the open- and closed-form DAE calculated at the B3LYP and ωB97X-D levels of theory using 6-31G(d) and 6-311++G(d,p) basis sets. All values are given in eV

Structure	VIP	AIP	VEA	AEA
**B3LYP/6-31G(d)**				
Open-DAE	+6.42	+6.21	−0.22	−0.02
Closed-DAE	+5.95	+5.74	+0.13	+0.41

**ωB97X-D/6-31G(d)**				
Open-DAE	+6.27	—	−0.27	—
Closed-DAE	+6.17	—	+0.11	—

**ωB97X-D/6-311++G(d,p)**				
Open-DAE	+6.70	—	+0.07	—
Closed-DAE	+6.31	—	+0.43	—

We also tested the effect of including diffuse functions in the basis set on the calculated electron affinities and ionization potentials. The results show a considerable increase in VEA and VIP (Table 1). Importantly, for the open-DAE, the VEA value changes from negative to slightly positive.

The absorption spectra of the free open- and closed-DAE molecules show clear differences between the two isomers (Fig. 2). At the TD-B3LYP level (Fig. 2a), the closed-DAE form exhibits an intense absorption band at 2.59 eV, together with additional bands at higher excitation energies. In contrast, the open-DAE form shows negligible absorption in the low-energy region and its main absorption band appears at higher energy, at 3.58 eV. This indicates that the closed form has a lower-energy optical response than the open form, which can be attributed to the more extended π-conjugation in the closed-ring structure.

**Fig. 2:**
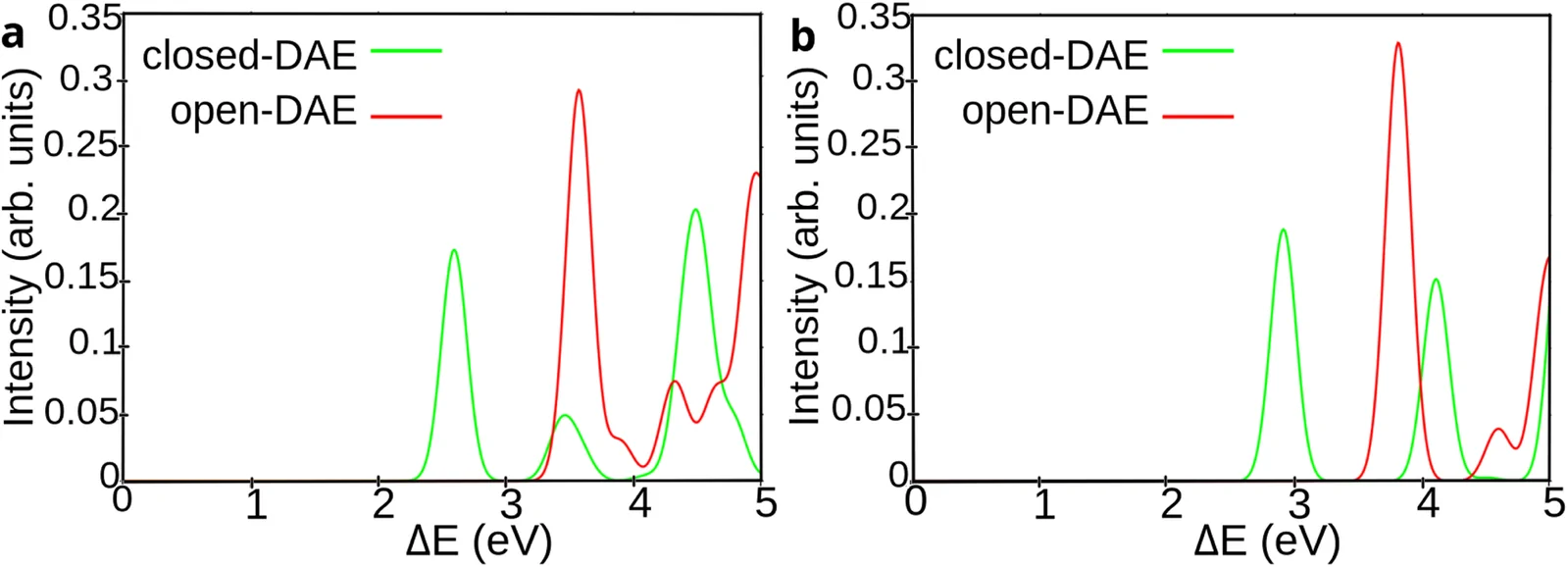
Calculated absorption spectra of the free open- and closed-DAE molecules in the absence of Au clusters: (a) TD-B3LYP and (b) TD-ωB97X-D.

A similar trend is observed at the TD-ωB97X-D level (Fig. 2b). The closed-DAE molecule again displays its first intense absorption band at lower energy than open-DAE, with a main feature at 2.91 eV and additional higher-energy peak near 4.11 eV. The open-DAE spectrum is dominated by a strong absorption band at 3.81 eV, while weaker transitions appear at higher energies. Therefore, both functionals predict that the closed-DAE isomer absorbs at lower excitation energies than the open-DAE isomer.

Overall, the spectra demonstrate the characteristic photoisomer-dependent optical behaviour of DAE. The closed-DAE form absorbs at lower energies, extending further into the visible region, whereas the open-DAE form mainly absorbs at higher energies, in the near-UV region. This difference between the two isomers is important for the photoswitching behaviour of DAE and provides a reference for evaluating how Au–cluster attachment modifies the electronic excitation spectrum.

### 3.2 Effect of Au_*n*_ clusters on the isomerization energetics

The structures of open- and closed-DAE + Au_10_ are shown in Fig. 1d and f, respectively, and the corresponding TS in Fig. 1e. The structures of the complexes with Au_20_ are shown in Fig. 1g–i. The Au_10_ cluster is a planar sheet, whereas Au_20_ is a tetrahedron. These gold clusters have been previously used in combination with other molecules in the context of plasmonic chemistry.^54–57^

Table 2 summarizes the relative energies of the open-form DAE, TS, and closed-form DAE for free DAE and DAE adsorbed on Au_10_ and Au_20_ clusters. For the free DAE, the energy barrier for the ring-closing pathway is relatively high, with values of 2.78 eV at the B3LYP level and 3.13 eV at the ωB97X-D level, measured relative to the open-form isomer. At the B3LYP level, the corresponding Gibbs free energy barrier is 2.68 eV, giving an extremely small rate constant of 3.10 × 10^−33^ s^−1^ at 298.15 K. The presence of a single imaginary frequency for each TS confirms that the optimized transition states correspond to first-order saddle points along the ring-opening/ring-closing reaction coordinate.

**Table 2:** Relative energies of the open-form DAE, transition state (TS), and closed-form DAE for the free molecule and for DAE adsorbed on Au_10_ and Au_20_ clusters. The electronic barrier Δ*E*^‡^, Gibbs free energy barrier Δ*G*^‡^, imaginary frequency of the TS, and rate constant *k* are reported. Energies are given in eV

Method	Open-DAE	TS (Δ*E*^‡^)	Closed-DAE	TS (Δ*G*^‡^)	Freq. (cm^−1^)	*k* (s^−1^)
**Free DAE (RDFT)**						
RB3LYP	0.0	+2.78	+0.65	+2.68	3119.59i	3.10 × 10^−33^
RωB97X-D	0.0	+3.13	+0.66	—	—	—

**Free DAE (BS-UDFT)**						
UB3LYP	0.0	+2.30	+0.65	+2.18	615.13i	7.75 × 10^−25^
UωB97X-D	0.0	+2.47	+0.66	—	—	—

**Free thiyl radical**						
UB3LYP	0.0	+1.50	+0.22	+1.47	425.84i	8.81 × 10^−13^
UωB97X-D	0.0	+1.76	+0.23	—	—	—

**DAE–Au**_**10**_						
UB3LYP	0.0	+1.47	+0.16	+1.45	424.27i	1.92 × 10^−12^
UωB97X-D	0.0	+1.75	+0.18	—	—	—

**DAE–Au**_**20**_						
UB3LYP	0.0	+1.65	+0.40	+1.58	483.98i	1.22 × 10^−14^
UωB97X-D	0.0	+1.78	+0.24	—	—	—

However, the imaginary frequency for the TS is unusually large, 3119.59i cm^−1^. We performed an SCF stability analysis for this TS and found that the wave function has a triplet instability. The free-DAE TS was then reoptimized at the broken-symmetry (BS) UB3LYP level. The BS-UB3LYP TS shows a single imaginary frequency of 615.13i cm^−1^.

Further, since the H atom of the SH group is removed in our hybrid DAE–gold models (see Fig. 1), we also performed calculations for the DAE thiyl radical. The corresponding energetics are presented in Table 2. It is seen that the activation energy is further reduced, Δ*G*^‡^ = 1.47 eV at the UB3LYP level. Also, the reaction energy is lower for the radical (0.22 eV) than for the parent DAE molecule with the H atom (0.65 eV).

These results indicate that thermal interconversion between the open- and closed-form isomers is highly unfavorable for the free parent molecule (with H). This finding is consistent with the well-known photochromic behaviour of diarylethenes, for which the isomerization is typically driven by light rather than by thermal activation. In general, UV irradiation induces ring-closing of the thermodynamically more stable open form, whereas visible light promotes the reverse ring-opening process.^4,5^

For comparison with available literature data, albeit for other diarylethenes,^58^ the closed-to-open thermal cycloreversion barrier was estimated from the energy difference between the TS and the closed-form DAE. The reported, experimental activation energies for the thermal cycloreversion of the closed-ring diarylethenes are in the range of 92–139 kJ mol^−1^, corresponding to approximately 0.95–1.44 eV.^58^ In the present work, the calculated cycloreversion barrier of the free (stiff) DAE is 2.13 eV at the B3LYP level and 2.47 eV at the ωB97X-D level (when using RDFT) and 1.65 eV at the B3LYP level and 1.81 eV at the ωB97X-D level (when using BS-UDFT).

Adsorption of DAE on gold clusters significantly modifies the reaction energetics. For DAE on Au_10_, the electronic barrier decreases to 1.47 eV at the B3LYP level and 1.75 eV at the ωB97X-D level. A similar trend is observed for the Au_20_ cluster, where the barrier is reduced to 1.65 eV at the B3LYP level and 1.78 eV at the ωB97X-D level. The corresponding rate constants are also much larger than that of the free DAE, indicating that the gold clusters facilitate the isomerization process. The unrestricted TS solutions for the open-to-closed DAE isomerization on Au_10_ and Au_20_ were verified by SCF stability analysis and were found to be stable.

The relative energetic stability of the closed-form DAE is also affected by interaction with the gold clusters. While the closed form of the free DAE lies about 0.65 eV above the open form at the B3LYP level, this energy difference decreases to 0.16 eV for DAE + Au_10_ and 0.40 eV for DAE + Au_20_.

The lowered isomerization barriers and reaction energies observed upon adsorption on gold clusters agree with the corresponding energies for the thiyl radical. Moreover, the spin density for hybrid systems is primarily localized on DAE (see SI, Sec. S3), supporting the molecular radical formation.

Finally, we note that for the free DAE, the relatively high closed-to-open barrier (2.13 eV with RB3LYP and 1.65 eV with BS-UB3LYP) is consistent with thermally stable, predominantly photochemical switching (P-type photochromism).^6,59^ Upon adsorption on Au_10_ and Au_20_, however, the calculated closed-to-open barriers decrease to 1.31 and 1.25 eV, respectively, at the UB3LYP level. This suggests that coupling to gold may facilitate thermal ring opening and shift the system toward the T-type photochromic behaviour.

### 3.3 Effect of Au_*n*_ clusters on the absorption spectra

The effect of Au_10_ attachment on the absorption spectra of open-DAE + Au_10_ and closed-DAE + Au_10_ hybrid systems is shown in Fig. 3. In both systems, the absorption spectra are changed compared with the free DAE molecules. At the TD-B3LYP level (Fig. 3a), the open-DAE + Au_10_ hybrid system exhibits several new absorption bands in the low-energy region, below the main absorption band of the free open-DAE molecule. These features appear mainly between about 1 and 2.5 eV and indicate that interaction with the Au_10_ cluster introduces additional low-energy electronic transitions. For the closed-DAE + Au_10_ hybrid system (Fig. 3b), low-energy and medium-energy bands are also observed, with appreciable intensity around 2.4–2.6 eV. Thus, Au_10_ attachment produces new spectral features that are absent in the free DAE spectra. We also note that, for open-DAE, the spectra obtained from 50 (or even 100 in the case of ωB97X-D, see below) lowest excited states calculated for the hybrid systems do not reach the lowest energy absorption band of free open-DAE.

**Fig. 3:**
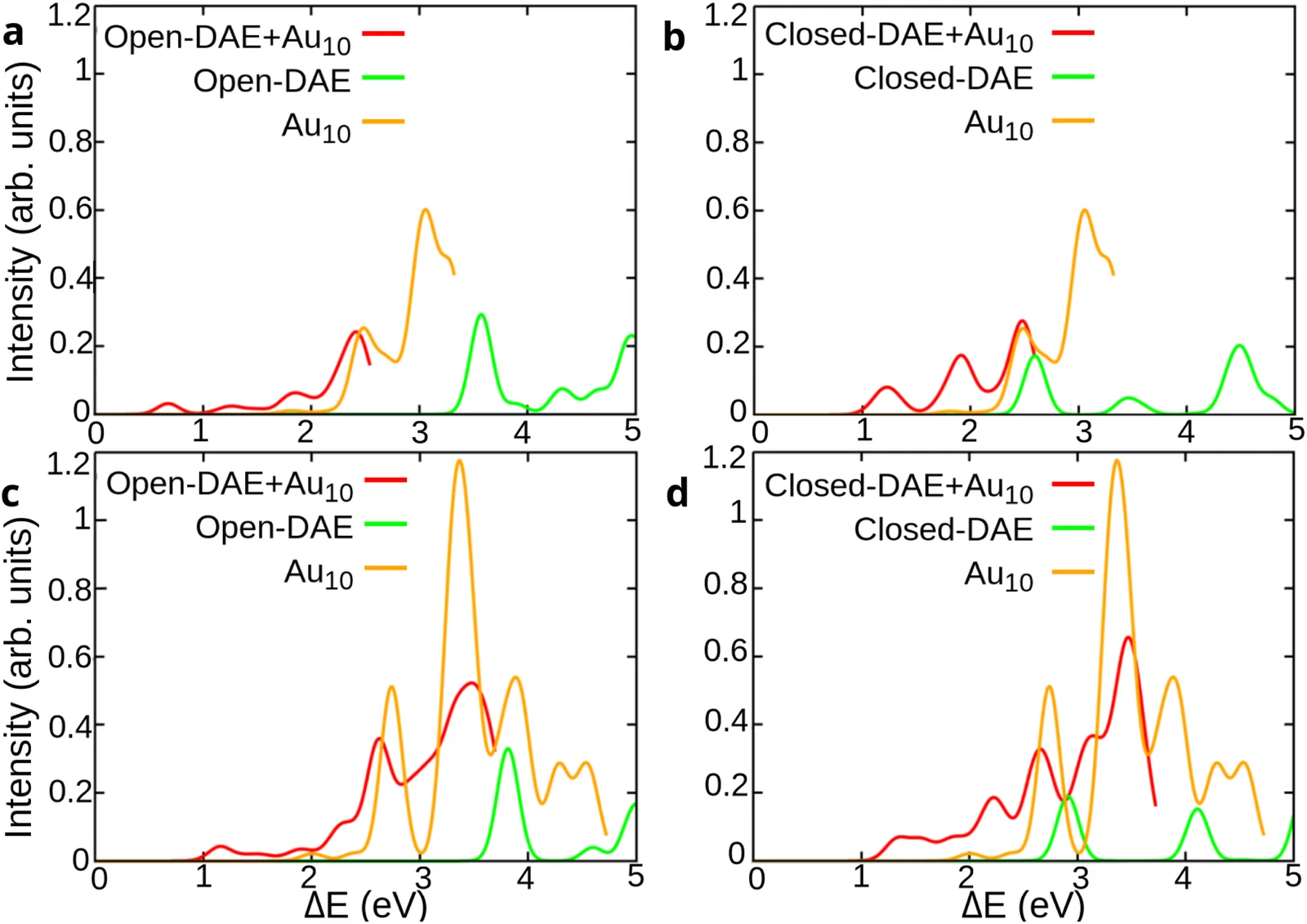
Absorption spectra of the free DAE molecular forms, open- and closed-DAE + Au_10_ hybrid systems, and the pure Au_10_ cluster calculated using B3LYP for panels (a and b) with 50 excited states and ωB97X-D for panels (c and d) with 100 excited states. Each spectrum for hybrid systems and gold cluster is truncated at the highest excitation energy included in the corresponding TD-DFT calculations.

The free Au_10_ cluster shows intense absorption bands in the energy range of approximately 2.5–3.5 eV at the TD-B3LYP level. These Au_10_-related transitions overlap with the molecular absorption region and also extend to the red of it. Importantly, the spectra of the DAE + Au_10_ hybrid systems cannot be described simply as a superposition of the free DAE and free Au_10_ spectra. Instead, the appearance of additional peaks and the change in intensity distribution suggest electronic coupling between the DAE molecule and the gold cluster.

It is known that B3LYP (a global hybrid functional with 20% exact exchange) can underestimate the energies of charge-transfer states;^60^ therefore, we have also performed calculations using ωB97X-D (a long-range corrected hybrid functional). In what follows, the TD-B3LYP results are presented alongside TD-ωB97X-D results for comparison.

At the TD-ωB97X-D level (Fig. 3c and d), the same general behaviour is observed, although the spectral features are shifted and their relative intensities change. The open-DAE + Au_10_ hybrid system again shows several absorption bands at lower energies than the main band of the free open-DAE molecule. Similarly, the closed-DAE + Au_10_ hybrid system displays a broadened absorption profile with multiple peaks between about 1.5 and 3.2 eV. The Au_10_ cluster itself exhibits intense bands at the TD-ωB97X-D level in the range around 2.5–4.0 eV.

Comparing the free and Au_10_-attached systems, the most important effect of the gold cluster is the appearance of additional absorption bands at lower excitation energies. In summary, the free DAE molecule shows a clear photoisomer-dependent optical response, with the closed form absorbing at lower energy than the open form. This behaviour is characteristic of diarylethene photochromes, for which ring closure extends the π-conjugation and shifts the absorption to lower energies, often into the visible region.^5^ After attachment to Au_10_, both isomers exhibit additional low-energy transitions and broader spectral profiles. The character of these transitions will be analyzed below, in Sec. 3.5.

To further examine the effect of cluster size, larger gold clusters, Au_19_ and Au_20_, were considered. Au_19_ was obtained from Au_20_ by removing one vertex atom as shown in Fig. S5d and S6d, which makes the whole hybrid formally a closed-shell system. As shown in Fig. 4, the spectra of the open- and closed-DAE + Au_19_ and open- and closed-DAE + Au_20_ systems differ markedly from those of the corresponding free DAE molecules.

**Fig. 4:**
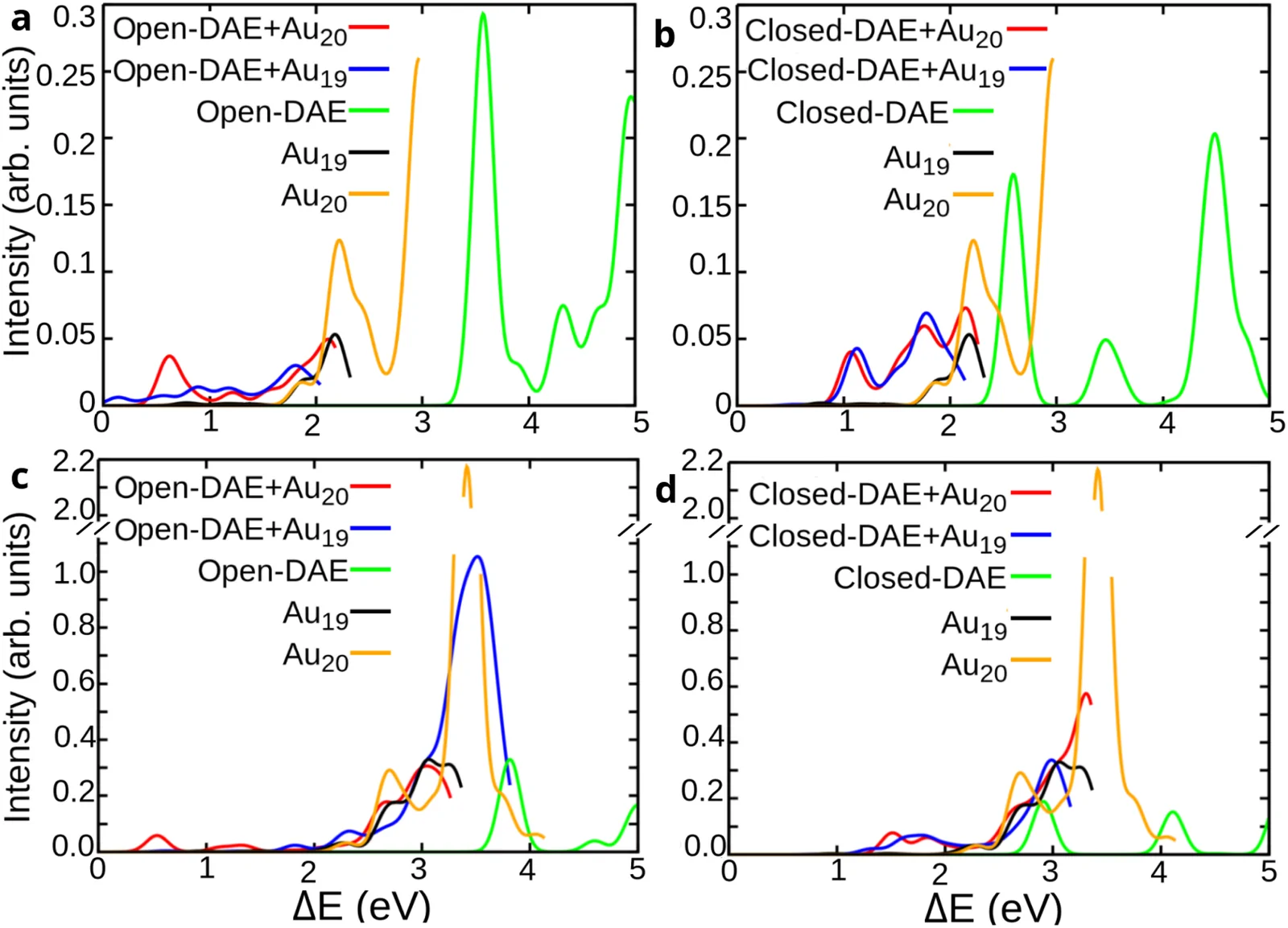
Absorption spectra of the closed and open systems with Au_19_ and Au_20_ clusters, together with the spectra of the free systems and pure Au_19_ and Au_20_ clusters calculated using B3LYP for panels (a and b) with 50 excited states and ωB97X-D for panels (c and d) with 100 excited states. Each spectrum for hybrid systems and gold cluster is truncated at the highest excitation energy included in the corresponding TD-DFT calculations.

At the TD-B3LYP level (Fig. 4a and b), the closed-DAE + Au_19_ and closed-DAE + Au_20_ spectra (Fig. 4b) show several weak-to-moderate bands in the range of about 1.0–2.2 eV, while the free closed-DAE absorption is dominated by the molecular band around 2.6 eV and by higher-energy features above 4 eV. For the open isomer (Fig. 4a), Au_19_ and Au_20_ attachment produces several weak low-energy absorption bands below 2.5 eV.

At the TD-ωB97X-D level (Fig. 4c and d), the intensity of the Au_20_ absorption becomes significantly larger. In contrast, Au_19_ produces weaker absorption features, mainly distributed between approximately 1.5 and 3.1 eV. Again, the onset of absorption of hybrid systems is red-shifted with respect to that of composing components (bare gold cluster and free molecule).

In a DFT-based theoretical study, Perrier *et al.* showed that connecting dithienylethene derivatives to small gold clusters, Au_3_ and Au_9_, strongly modifies the absorption spectrum of the open-ring isomer and may thereby inhibit the ring-closure process, whereas the electronic transition associated with ring opening in the closed-ring isomer is largely preserved.^25^ This interpretation is consistent with the experimental findings of Dulić *et al.*, who observed one-way optoelectronic switching of dithienylethene molecules on gold:^61^ the closed-ring conducting form could be converted to the open-ring insulating form, whereas the reverse ring-closing process was suppressed.

This interpretation is also consistent with the DFT/TD-DFT study of Fihey *et al.* on DTE + Au_25_ hybrid switches, where the optical response was shown to depend on both the photochrome π-system and the electronic states of the gold nanocluster.^62^ Similarly, the additional low-energy bands observed here for DAE + Au_10_, DAE + Au_19_, and DAE + Au_20_ indicate that the gold clusters actively participate in the excited-state spectrum rather than acting only as inert supports.

### 3.4 Electronic stability and optical (S_0_–S_1_) gaps of DAE–Au_*n*_ hybrid systems

Table 3 summarizes the electronic (self-consistent field, SCF) stability, first excitation energies or optical gaps *E*(S_1_) [we note that some S_0_ → S_1_ transitions have rather small oscillator strengths; yet, we call the S_0_–S_1_ energy difference the “optical gap” for simplicity; moreover, for the doublet systems “S” means “state”, not “singlet”], oscillator strengths *f*(S_1_), and spin-contamination indicators 〈*S*^2^〉 (the expectation value of squared spin operator) for the free DAE molecules and the DAE–Au_*n*_ hybrid systems. The checkmark (✓) in the “stability” column means that the SCF (Kohn–Sham) solution is stable, whereas numbers in this column show the energy difference (in eV) between the unstable and stable SCF solutions. The TD-DFT calculations were performed on the stable SCF references.

**Table 3:** Stability, optical gaps *E*(S_1_), oscillator strengths *f*(S_1_) and spin-contamination indicator 〈*S*^2^〉 for free and adsorbed DAE on Au_10_, Au_19_, and Au_20_ clusters. Energies are given in eV. For the stability analysis, ✓ indicates a stable SCF solution, whereas a number provides energy difference (in eV) with respect to a stable solution

Method	Stability	*E*(S_1_)	*f*(S_1_)	〈*S*^2^〉
**Free open-form DAE**				
B3LYP	✓	3.5718	0.2928	0.0
ωB97X-D	✓	3.8134	0.3295	0.0

**Free closed-form DAE**				
B3LYP	✓	2.5941	0.1732	0.0
ωB97X-D	✓	2.9105	0.1893	0.0

**Open-form DAE on Au**_**10**_				
B3LYP	✓	0.6772	0.0310	0.801
ωB97X-D	✓	1.0054	0.0015	0.912

**Closed-form DAE on Au**_**10**_				
B3LYP	✓	1.0115	0.0019	1.062
ωB97X-D	✓	1.2216	0.0037	2.477

**Open-form DAE on Au**_**19**_				
B3LYP	0.047	0.1366	0.0061	0.351
ωB97X-D	✓	0.6282	0.0038	0.0

**Closed-form DAE on Au**_**19**_				
B3LYP	0.321	0.5034	0.0005	0.396
ωB97X-D	0.654	0.8645	0.0013	1.062

**Open-form DAE on Au**_**20**_				
B3LYP	✓	0.6113	0.0345	0.766
ωB97X-D	✓	0.5429	0.0582	0.786

**Closed-form DAE on Au**_**20**_				
B3LYP	✓	1.0642	0.0394	0.843
ωB97X-D	✓	1.5064	0.0728	0.959

The free open- and closed-form DAE molecules are electronically stable closed-shell systems, as indicated by 〈*S*^2^〉 = 0.0. The open form shows a larger optical gap, with *E*(S_1_) values of 3.57 eV at the B3LYP level and 3.81 eV at the ωB97X-D level. In contrast, the closed form has lower *E*(S_1_) values of 2.59 eV (B3LYP) and 2.91 eV (ωB97X-D), respectively.

After adsorption on gold clusters, the first excitation energies are strongly reduced. For DAE + Au_10_, the *E*(S_1_) values decrease to 0.68 eV (1.01 eV) for the open form and 1.01 eV (1.22 eV) for the closed form, for B3LYP (ωB97X-D). Similar low-energy excitations are found for DAE + Au_19_ and DAE + Au_20_. In particular, DAE + Au_19_ gives very small optical gaps, especially at the B3LYP level. We note that for the bare Au_10_ cluster, the S_0_–S_1_ gap is 1.59 eV (1.86 eV), and for the bare Au_20_ cluster, 1.79 eV (2.14 eV) at the B3LYP (ωB97X-D) level. These results indicate that coupling to gold clusters introduces new low-energy electronic states that are absent in the free DAE molecule, at least at the used levels of theory. Interestingly, for the hybrid systems, the closed form has a higher S_1_ excitation energy than the open form.

The nonzero 〈*S*^2^〉 values (for the nominally closed-shell systems) or deviating from 0.75 (for the nominally doublet, open-shell systems), observed for several DAE–Au_*n*_ hybrids, indicate spin contamination, which is likely related to the electronic structure of the gold clusters and molecule–metal coupling. Nevertheless, the same general trend is observed for both B3LYP and ωB97X-D: adsorption on gold clusters significantly lowers the optical gap. The small oscillator strengths, *f*(S_1_), suggest that the lowest-energy excitations are weakly optically allowed and may involve some charge transfer.

We have also examined further models, such as with additional H atom adsorbed on Au_20_ (which makes the whole hybrid formally a closed-shell system) or optimized without D3 correction (which yields different molecular orientations), see Fig. S5 and S6. The optical gaps and results of stability analysis are provided in Tables S1 and S2. These models also show small optical gaps of ≲1 eV.

### 3.5 FTDM analysis—charge transfer and local excitations in DAE–Au_*n*_ hybrid systems

Fig. 5 and 6 show the FTDM analysis for the open- and closed-DAE + Au_10_ hybrid systems calculated at the ωB97X-D level. Accordingly, all assignments and discussion of CT states in the following are based on the ωB97X-D results. In the FTDM analysis, the hybrid metal–molecule system is partitioned into two fragments—the gold cluster (G) and the DAE molecule (M)—and the 2 × 2 FTDM matrix, eqn (6), is computed, for transition 0 → *I*. We note that the linking S atom was counted in the M fragment. The *F*_11_ matrix element quantifies the contribution of the local molecular excitation (M → M), whereas *F*_22_ shows the contribution of the local gold excitation (G → G). In what follows, we will call the states with relatively large *F*_11_ molecular states (MS) and those with rather large *F*_22_ gold states (GS) [not to be confused with a ground state]. The off-diagonal matrix elements describe charge-transfer (CT) character between the DAE molecule and the Au_10_ cluster, corresponding either to M → G (*F*_12_) or G → M (*F*_21_) charge transfer.

**Fig. 5:**
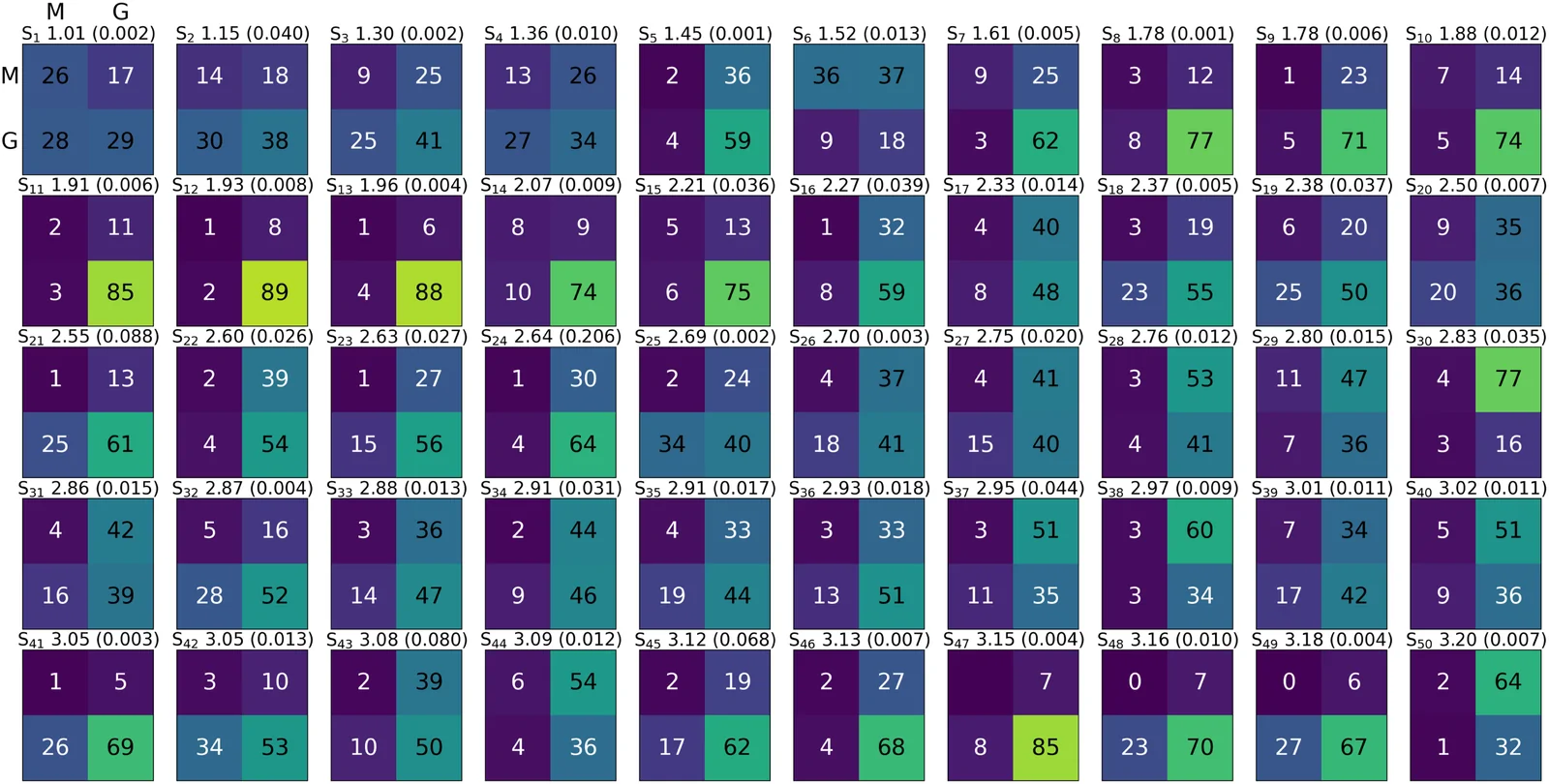
The FTDM matrices for open-DAE + Au_10_ at the ωB97X-D level. M and G letters refer to DAE molecule and gold, respectively. S_1_ to S_50_ refer to excited state numbers from 1 to 50, the value after state number is the transition energy in eV, and the value in parentheses is the oscillator strength of the transition from the ground state.

**Fig. 6:**
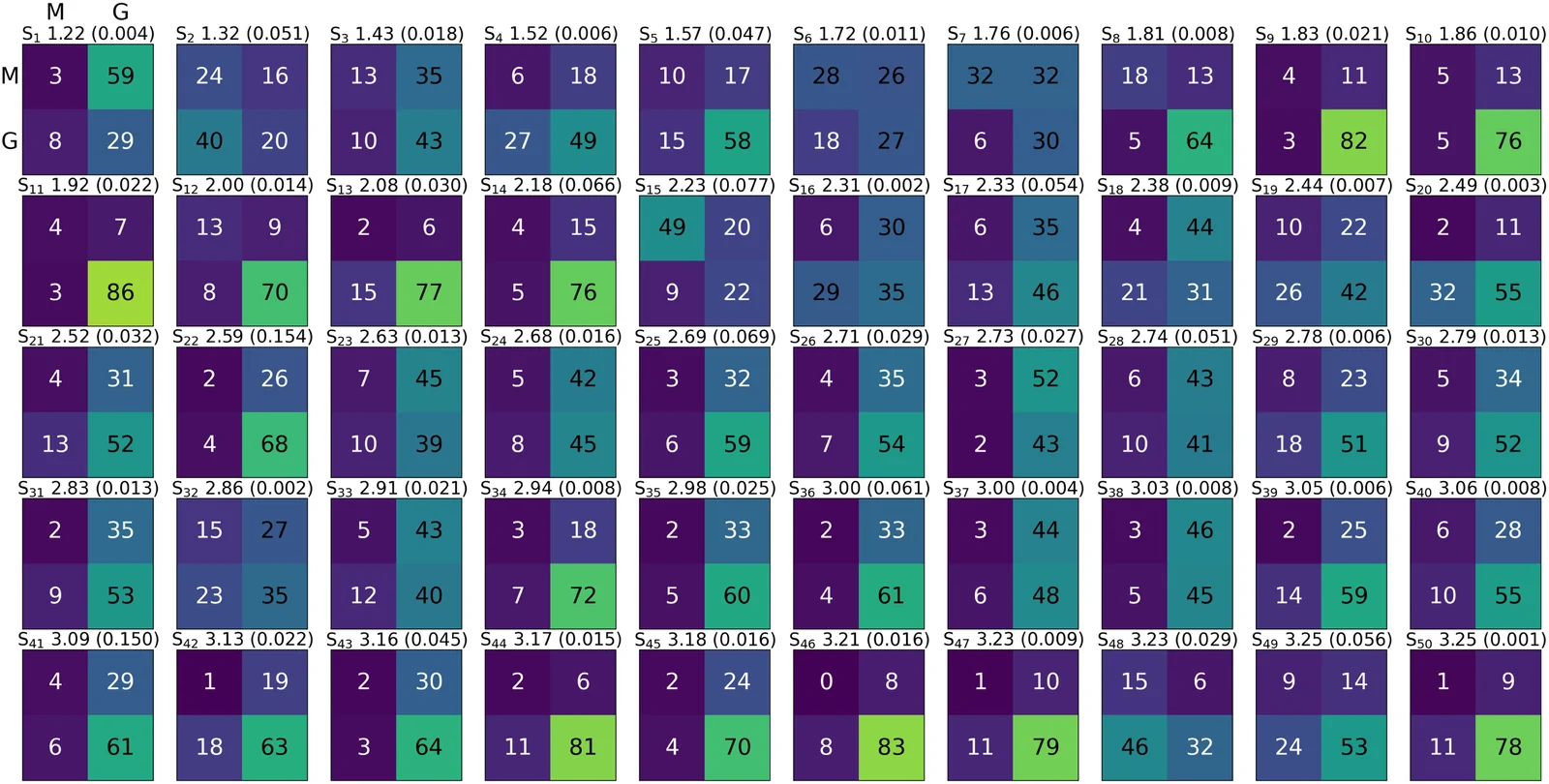
The FTDM matrices for closed-DAE + Au_10_ at the ωB97X-D level. M and G letters refer to DAE molecule and gold, respectively. S_1_ to S_50_ refer to excited state numbers from 1 to 50, the value after state number is the transition energy in eV, and the value in parentheses is the oscillator strength of the transition from the ground state.

For the open-DAE + Au_10_ hybrid system (Fig. 5), the dominant M → G CT state is observed for the S_0_ → S_30_ transition at 2.83 eV, with a CT contribution of 77%. A rather large G → M CT contribution (34%) is found for the S_0_ → S_25_ transition at 2.69 eV. The most prominent GS is S_12_ at 1.93 eV, with an Au_10_ contribution of 89%. A rather large molecular contribution is found for the S_0_ → S_6_ transition at 1.52 eV, where the DAE-localized contribution is 36%; however, this state also contains 37% of M → G CT. Nevertheless, we term this state “MS”. The oscillator strengths of these selected transitions are 0.035 for CT(S_30_), 0.002 for CT(S_25_), 0.008 for GS(S_12_), and 0.013 for MS(S_6_), indicating that these transitions are weakly optically active. Fig. S1 displays the hole and particle NTOs for the S_30_, S_25_, S_12_, and S_6_ states for the open-DAE + Au_10_ hybrid system. We also note that the lowest excited states S_1_–S_4_ are mixtures of all four excitation types (2 LEs and 2 CTs), *i.e.*, all four FTDM elements are quite substantial.

For the closed-DAE + Au_10_ hybrid system (Fig. 6), the dominant M → G CT state corresponds to the S_0_ → S_1_ transition at 1.22 eV, with a CT contribution of 59%. The dominant G → M CT state is associated with the S_0_ → S_2_ transition at 1.32 eV, with a contribution of 40%. The most pronounced GS is S_11_ at 1.92 eV, with an Au_10_ contribution of 86%, whereas we assign S_15_ as MS, with a DAE-localized contribution of 49%. The corresponding oscillator strengths are again small: 0.004 for CT(S_1_), 0.051 for CT(S_2_), 0.022 for GS(S_11_), and 0.077 for MS(S_15_). Fig. S2 displays the hole and particle NTOs of S_1_, S_2_, S_11_, and S_15_ states for the closed-DAE + Au_10_ hybrid system.

For the open-DAE + Au_20_ hybrid system (Fig. 7), the lowest-energy state with a dominant M → G charge-transfer character is the S_0_ → S_9_ transition at 2.0 eV, with an M → G contribution of 37%. However, the major contribution for this transition comes from the GS, 60%. This is reflected in the corresponding NTOs shown in Fig. S3—the NTOs are primarily localized on Au_20_. The reverse G → M charge-transfer character is found in the S_0_ → S_7_ transition at 1.77 eV, with a contribution of 54%, and also in the S_0_ → S_6_ (52%) and S_0_ → S_4_ (53%) transitions.

**Fig. 7:**
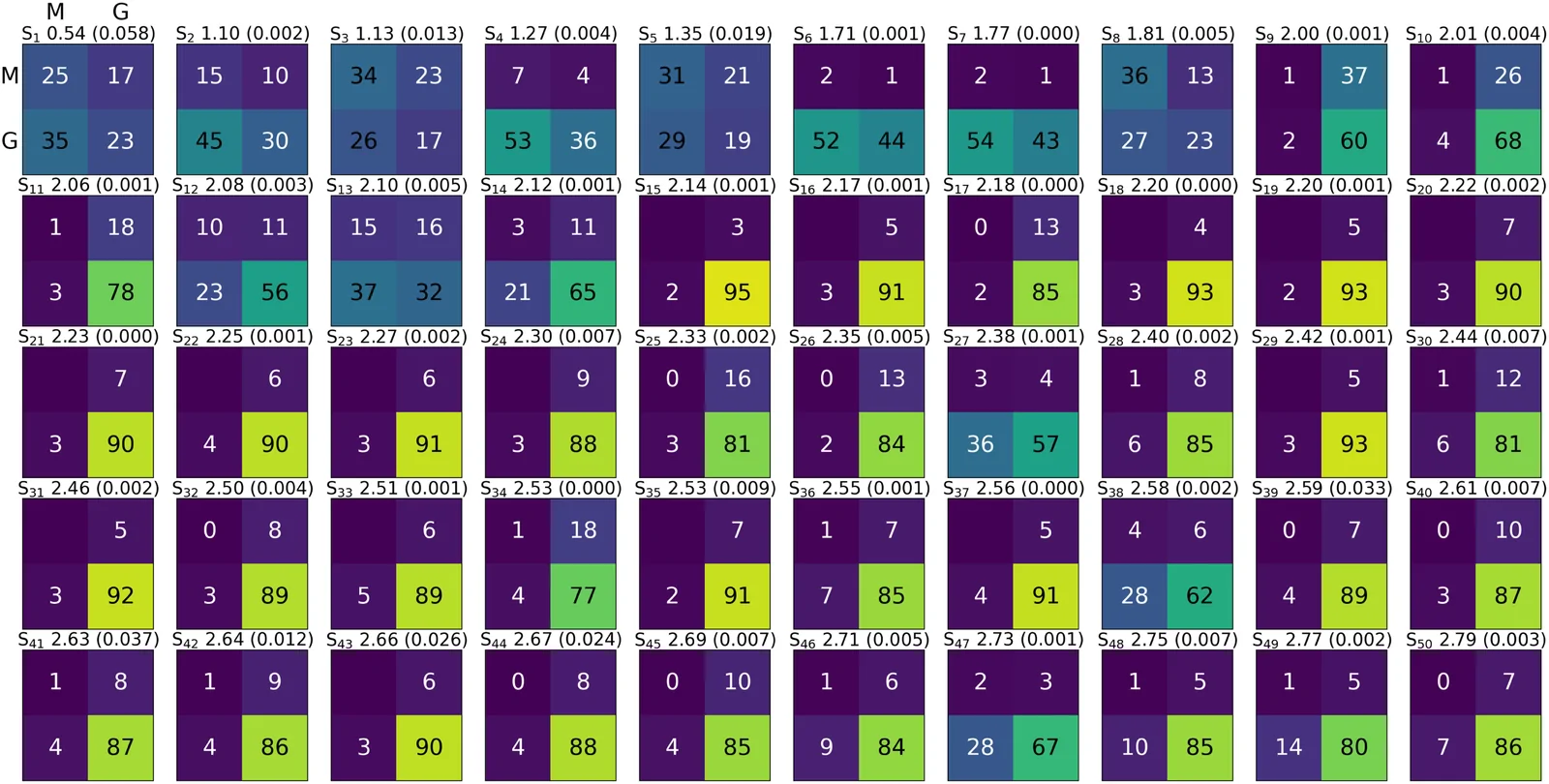
The FTDM matrices for open-DAE + Au_20_ at the ωB97X-D level. M and G letters refer to DAE molecule and gold, respectively. S_1_ to S_50_ refer to excited state numbers from 1 to 50, the value after state number is the transition energy in eV, and the value in parentheses is the oscillator strength of the transition from the ground state.

The major GS, localized on Au_20_, is state S_15_ at 2.14 eV, with a G → G contribution of 95%. The excitation predominantly localized on the DAE molecule (MS) is mainly associated with the S_0_ → S_8_ transition at 1.81 eV, with an M → M contribution of 36%. The other FTDM matrix elements are, however, also quite substantial (between 10 and 30%) for this transition. The corresponding oscillator strengths are 0.001 for S_9_, 0.000 for S_7_, 0.005 for S_8_, and 0.001 for S_15_. Fig. S3 displays the hole and particle NTOs of S_9_ (CT), S_7_ (CT), S_8_ (MS), and S_15_ (GS) states for the open-DAE + Au_20_ hybrid system.

For the closed-DAE + Au_20_ hybrid system (Fig. 8), the lowest-energy state with a dominant M → G charge-transfer character is S_6_ at 1.95 eV, with an M → G contribution of 38%. The reverse G → M charge-transfer character is found in the S_0_ → S_5_ transition at 1.94 eV, with a contribution of 50%. A slightly larger G → M contribution of 51% occurs for the S_0_ → S_22_ transition at 2.26 eV. Also, the S_1_ state exhibits a considerable G → M CT of 45%.

**Fig. 8:**
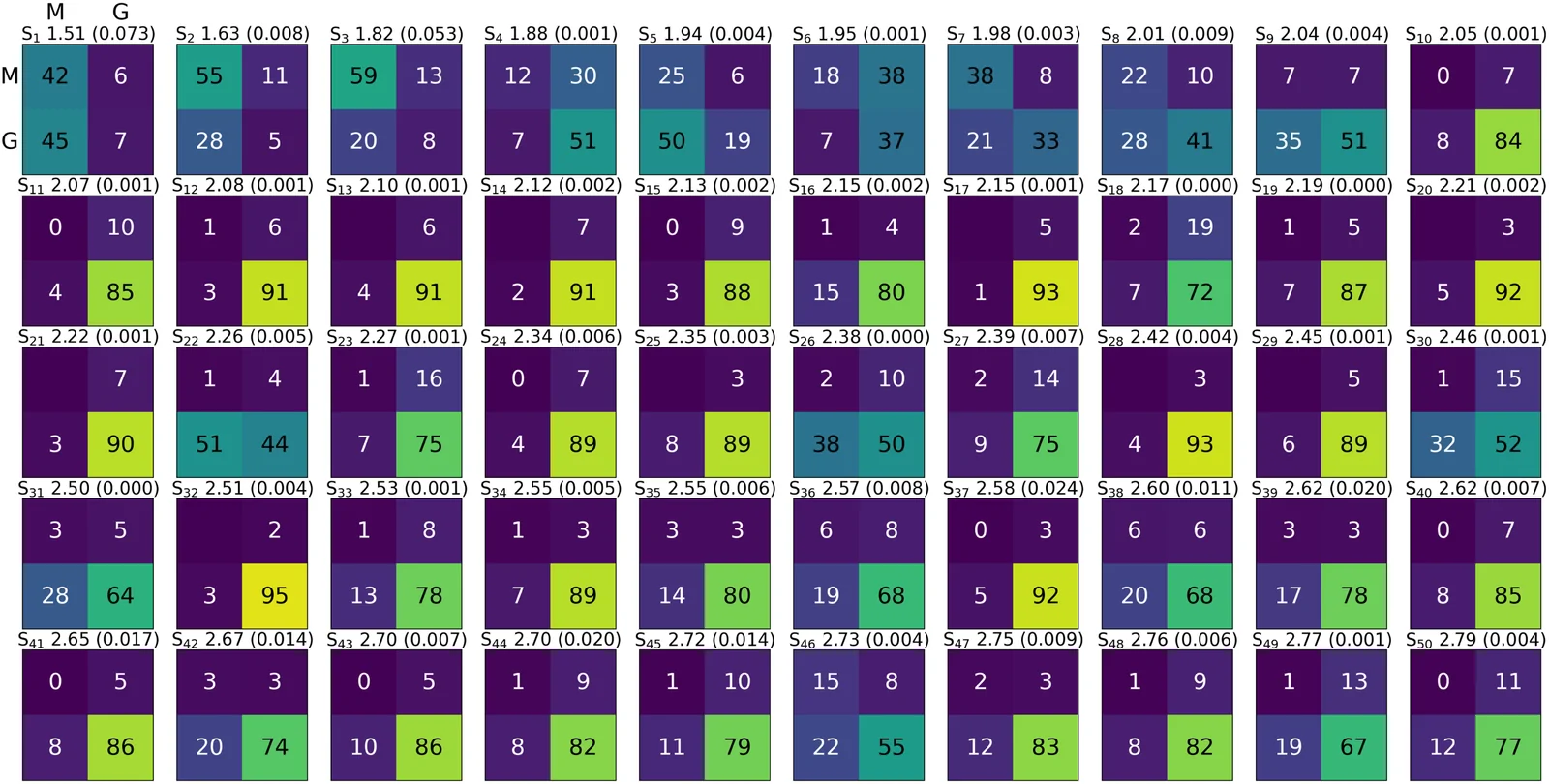
The FTDM matrices for closed-DAE + Au_20_ at the ωB97X-D level. M and G letters refer to DAE molecule and gold, respectively. S_1_ to S_50_ refer to excited state numbers from 1 to 50, the value after state number is the transition energy in eV, and the value in parentheses is the oscillator strength of the transition from the ground state.

Among the first 20 transitions, the dominant GS is S_17_ at 2.15 eV, with a G → G contribution of 93%. The largest Au_20_-localized contribution, 95%, is found for the S_0_ → S_32_ transition at 2.51 eV. The excitation predominantly localized on the DAE molecule (MS) is mainly associated with the S_0_ → S_3_ transition at 1.82 eV, with an M → M contribution of 59%. In addition, S_2_ has a 55% M → M contribution.

The corresponding oscillator strengths are 0.001 for S_6_, 0.004 for S_5_, 0.005 for S_22_, 0.001 for S_17_, 0.004 for S_32_, and 0.053 for S_3_. Among these states, the DAE-localized S_3_ excitation has the largest oscillator strength. Fig. S4 displays the hole and particle NTOs of S_3_ (MS), S_17_ (GS), S_6_ (CT) and S_5_ (CT) for the closed-DAE + Au_20_ hybrid system.

Furthermore, the FTDM matrices shown in Fig. 5–8 suggest that the low-energy excitations of the (stiff) DAE + Au_*n*_ hybrid systems have mixed character, involving both CT and LE (MS and GS). This finding is consistent with previous theoretical studies showing that gold clusters can strongly modify the optical response and excited-state character of (normal) dithienylethene–gold hybrid systems.^25,26,62^

The low-lying mixed and gold states are expected to participate in internal conversion and influence the efficiency of photoisomerization reactions. However, nonadiabatic, excited-state molecular dynamics simulations would be required to answer this question.^63^ Moreover, the presence of CT excitations indicates that photoinduced interfacial charge transfer is, in principle, electronically accessible in the DAE–Au hybrids. However, determining the efficiency and time scale of photoinduced charge transfer would again require excited-state dynamics, which goes beyond the scope of this work.

## 4 Conclusions

This study presents a density functional theory (DFT) and time-dependent density functional theory (TD-DFT) investigation of the ground-state reaction energetics and electronically excited states of novel diarylethene (DAE) photoswitch pcDTE_656_ (designed in ref. 23) chemisorbed on gold clusters, namely Au_10_, Au_19_, and Au_20_.

The calculations show that ring closure substantially changes the optical properties of the free DAE molecule by extending its π-conjugation, lowering the energy of its first absorption band, and making oxidation and electron attachment more favorable. Coupling DAE to gold nanoclusters greatly lowers ground-state isomerization barrier relative to the free molecule (through the formation of a molecular radical), leading to larger estimated thermal rate constants for the interconversion process. Adsorption on gold also decreases the energetic separation between the open and closed forms.

Furthermore, the metal cluster–molecule interaction introduces additional low-energy electronic transitions, appearing below 3 eV, with the S_0_–S_1_ gaps found near or even below 1 eV. The fraction of transition density matrix (FTDM) analysis reveals that the low-lying excited states have mixed character, including molecular, gold-cluster-localized, and bidirectional charge-transfer (CT) contributions. The role of these states in isomerization reactions remains to be elucidated.

Taken together, the results demonstrate that gold nanoclusters actively reshape the electronic and photochemical landscape of the DAE photoswitch by altering isomer stability, lowering the isomerization barrier, and introducing low-energy hybrid excited states.

## Author contributions

Elham Mazarei: data curation, formal analysis, investigation, methodology, software, visualization, writing – original draft, writing – review & editing. Evgenii Titov: conceptualization, formal analysis, funding acquisition, investigation, methodology, project administration, software, supervision, validation, writing – original draft, writing – review & editing.

## Conflicts of interest

There are no conflicts to declare.

## Supplementary Material

RA-016-D6RA07292E-s001

## Data Availability

Data for this article are available at Zenodo at https://doi.org/10.5281/zenodo.21823536. Supplementary information (SI): NTO analysis, further studied DAE–Au_*n*_ models, their optical gaps, and electronic stability data. See DOI: https://doi.org/10.1039/d6ra07292e.
