# Supplementary material for: Electronic states of diarylethene switch–gold nanocluster hybrids
Source: RSC Adv. 2026 Oct 30;16(49):67273–85. doi: 10.1039/d6ra07292e (PMC13720988; doi:10.1039/d6ra07292e)
Supplement: Supplementary file 1 [file RA-016-D6RA07292E-s001.pdf]

Supplementary Information (SI) for RSC Advances.
This journal is © The Royal Society of Chemistry

Supporting Information for "Electronic states of diarylethene switch–gold nanocluster hybrids"

Elham Mazarei* and Evgenii Titov*

Universität Potsdam, Institut für Chemie, Karl-Liebknecht-Str. 24-25, 14476 Potsdam, Germany

E-mail: mazarei@uni-potsdam.de; titov@uni-potsdam.de

Contents

S1 NTO Analysis **S2**

S2 Electronic Stability and Optical (S_0–S_1) Gaps of DAE–Au$_n$ Hybrid Systems **S4**

S3 Spin density **S7**

S1 NTO Analysis

Figures S1–S4 show dominant NTO hole–particle pairs for selected MS, GS, and CT states identified using the FTDM analysis for the DAE+Au_{10} and DAE+Au_{20} hybrid systems. The NTOs provide a qualitative visualization that complements the FTDM results. We note that for states with relatively small contribution of a leading NTO pair, this single NTO pair does not provide a complete representation of the excitation. However, state classification is performed using the FTDM analysis which treats the full TDM matrix (see Figures 5–8 in the main text).

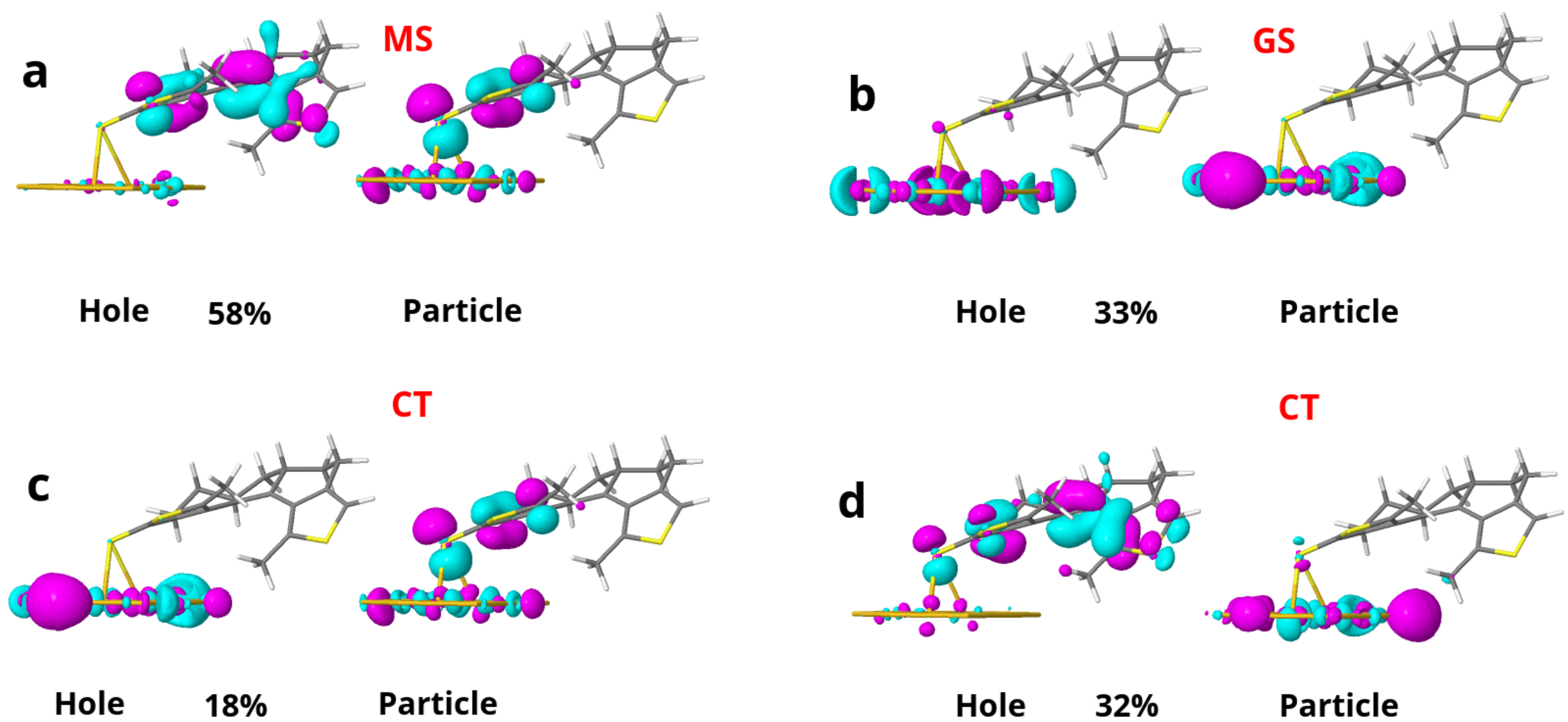

Figure S1: Hole and particle NTOs for the open-DAE+Au_{10} hybrid system obtained at the ωB97X-D level. The percentages shown in the figure indicate the contributions of the corresponding NTO pairs. Panel **a** displays MS (S_6, $F_{11} = 36\%$), panel **b** displays GS (S_{12}, $F_{22} = 89\%$), and panels **c** and **d** display the CT states (S_{25}, $F_{21} = 34\%$ and S_{30}, $F_{12} = 77\%$.)

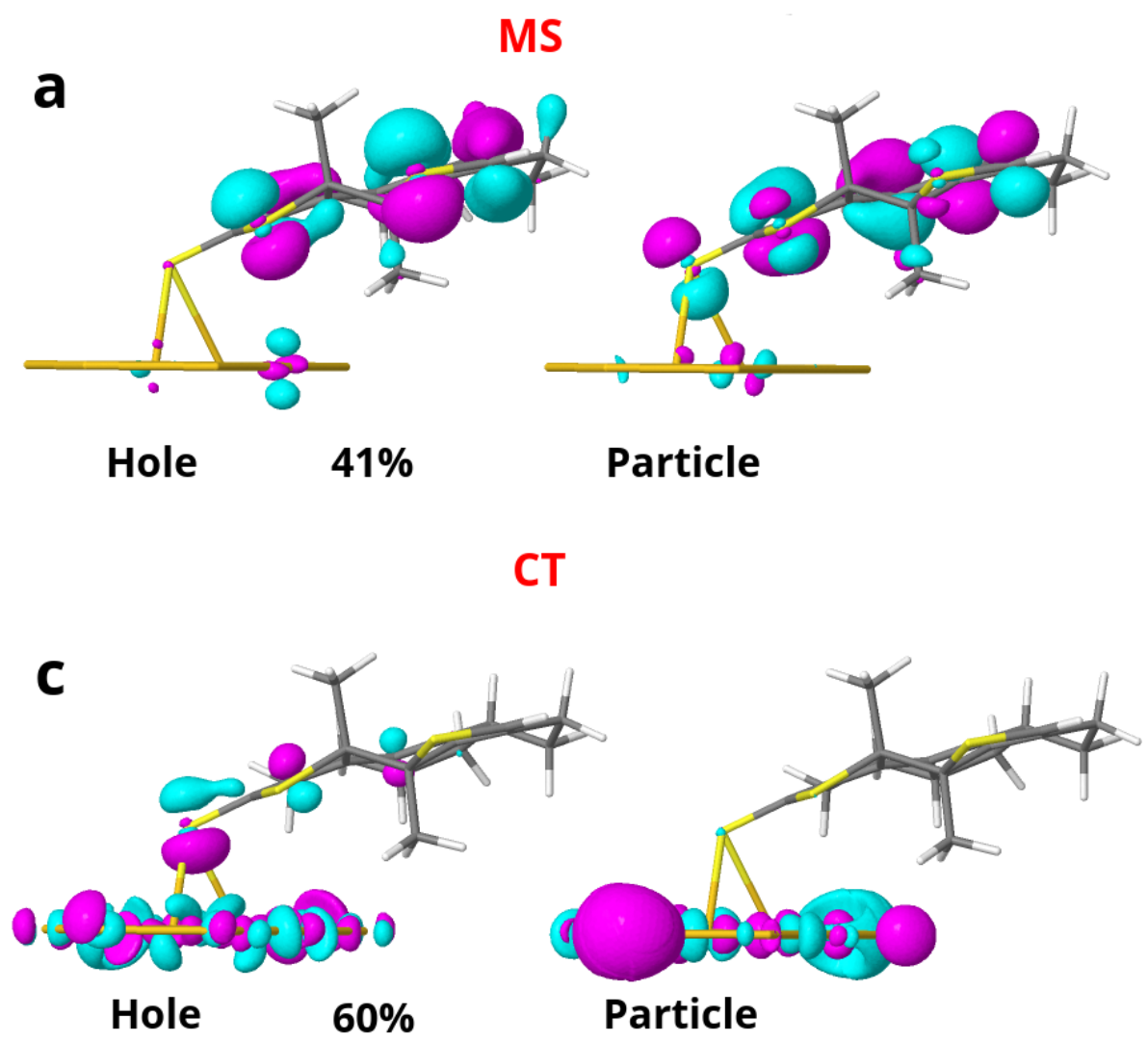

Figure S2: Hole and particle NTOs for closed-DAE+Au_{10} hybrid system obtained at the ωB97X-D level. The percentages shown in the figure indicate the contributions of the corresponding NTO pairs. Panel **a** display MS (S_{15}, $F_{11} = 49\%$), panel **b** displays the GS (S_{11}, $F_{22} = 86\%$) for Au_{10}, and panels (**c** and **d**) display the charge transfer states (S_1, $F_{12} = 59\%$ and S_2, $F_{21} = 40\%$).

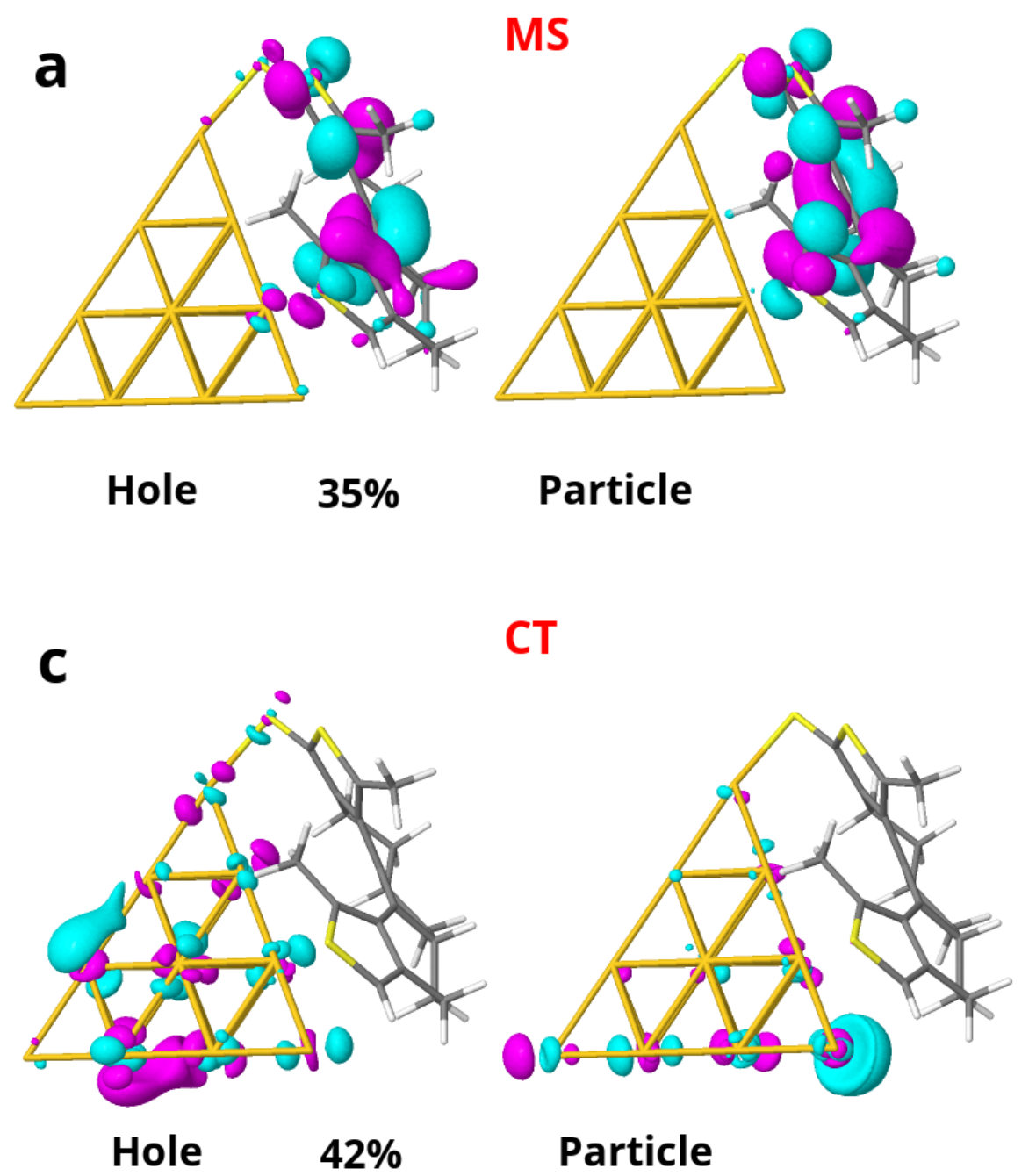

Figure S3: Hole and particle NTOs for open-DAE+Au_{20} hybrid system obtained at the ωB97X-D level. The percentages shown in the figure indicate the contributions of the corresponding NTO pairs. Panel **a** display MS (S_8, $F_{11} = 36\%$), panel **b** displays the GS (S_{15}, $F_{22} = 95\%$) for Au_{20}, and panels (**c** and **d**) display the charge transfer states (S_9, $F_{12} = 37\%$ and S_7, $F_{21} = 54\%$).

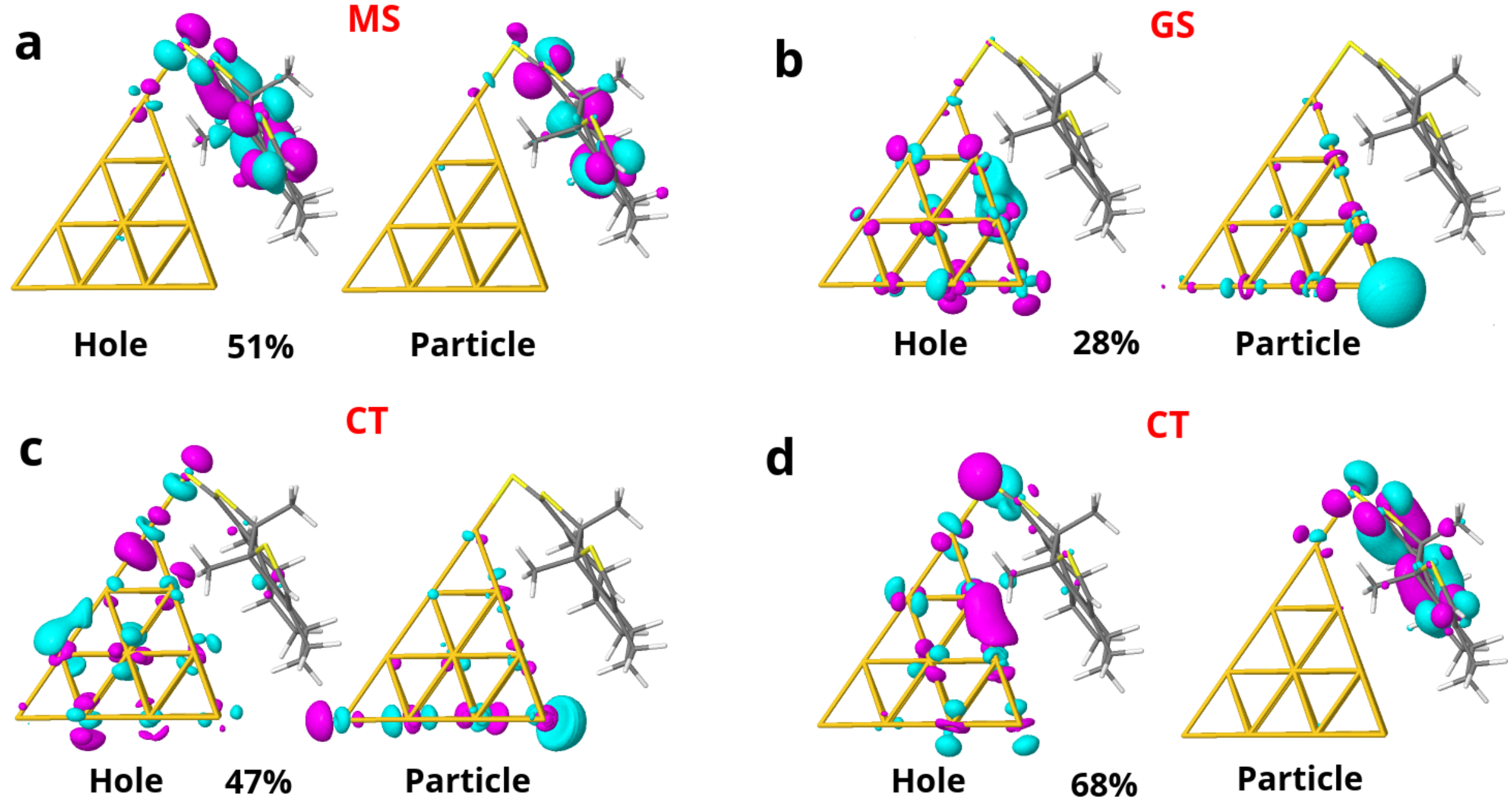

Figure S4: Hole and particle NTOs for closed-DAE+Au_{20} hybrid system obtained at the ωB97X-D level. The percentages shown in the figure indicate the contributions of the corresponding NTO pairs. Panel **a** display MS (S_3, $F_{11} = 59\%$), panel **b** displays the GS (S_{17}, $F_{22} = 93\%$) for Au_{20}, and panels (**c** and **d**) display the charge transfer states (S_6, $F_{12} = 38\%$ and S_5, $F_{21} = 50\%$).

S2 Electronic Stability and Optical (S_0–S_1) Gaps of DAE–Au_n Hybrid Systems

Tables S1 and S2 summarize the calculated electronic stability, optical gaps ($E(S_1)$), oscillator strengths ($f(S_1)$) and spin-contamination indicators ($\langle S^2 \rangle$) values for additional open- and closed-DAE–Au_n configurations. The results show that the optical gaps and spin character depend strongly on the gold-cluster model, adsorption geometry, and computational method.

Table S1: Stability, $E(S_1)$, $f(S_1)$, and $\langle S^2 \rangle$ for the open-form DAE adsorbed on different gold cluster configurations, calculated using various quantum chemical methods. All energy values are reported in eV. For the stability analysis, ✓ indicates a stable SCF solution, whereas a number provides energy difference (in eV) with respect to a stable solution.

Method	Stability	$E(S_1)$	$f(S_1)$	$\langle S^2 \rangle$
open-DAE+H+Au_{20}				
B3LYP	✓	0.6240	0.0014	0.0
ωB97X-D	✓	1.4615	0.0154	0.0
open-DAE+H+Au_{20}, no D3				
B3LYP	✓	0.7338	0.0018	0.0
ωB97X-D	✓	1.4735	0.0176	0.0
open-DAE+H+Au_{20}, no D3, upright				
B3LYP	✓	0.4229	0.0233	0.0
ωB97X-D	✓	1.3572	0.0350	0.0
open-DAE+Au_{19}				
B3LYP	0.047	0.1366	0.0061	0.351
ωB97X-D	✓	0.6282	0.0038	0.0
HF	0.723	0.4527	0.0086	2.512
CIS	see HF	0.7604	0.0084	2.575
open-DAE+Au_{20}				
B3LYP	✓	0.6113	0.0345	0.766
ωB97X-D	✓	0.5429	0.0582	0.786
HF	0.733[a]	0.4704	0.1012	2.245
CIS	see HF	0.8995	0.0733	2.233

[a]internal (UHF→UHF) instability

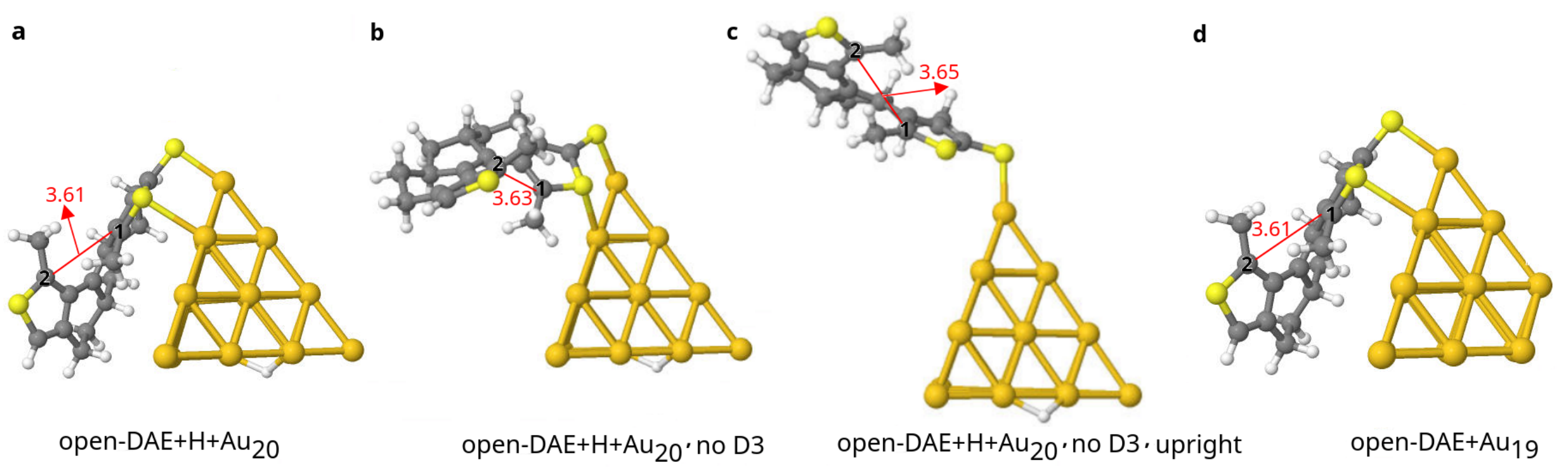

Figure S5: Additional models of open-form DAE adsorbed on Au_{19} and Au_{20} clusters considered in Table S1. Geometry optimizations were perfromed with B3LYP[+D3(BJ)].

Table S2: Stability, $E(S_1)$, $f(S_1)$, and $\langle S^2 \rangle$ for the closed-DAE adsorbed on different gold cluster configurations, calculated using various quantum chemical methods. All energy values are reported in eV. For the stability analysis, ✓ indicates a stable SCF solution, whereas a number provides energy difference (in eV) with respect to a stable solution.

Method	Stability	$E(S_1)$	$f(S_1)$	$\langle S^2 \rangle$
closed-DAE+H+Au_{20}				
B3LYP	0.045	0.1843	0.0104	0.576
ωB97X-D	✓	0.8862	0.0095	0
closed-DAE+H+Au_{20}, no D3				
B3LYP	✓	0.6517	0.0080	0
ωB97X-D	✓	1.3605	0.0136	0
closed-DAE+H+Au_{20}, no D3, upright				
B3LYP	0.022	0.2286	0.0084	1.015
ωB97X-D	✓	1.2253	0.0942	0
closed-DAE+Au_{19}				
B3LYP	0.321	0.5034	0.0005	0.396
ωB97X-D	0.654	0.8645	0.0013	1.062
HF	0.834	0.3483	0.0066	2.521
CIS	see HF	0.6922	0.0071	2.522
closed-DAE+Au_{20}				
B3LYP	✓	1.0642	0.0394	0.843
ωB97X-D	✓	1.5064	0.0728	0.959
HF	✓	1.4207	0.0702	2.148
CIS	see HF	1.8841	0.0601	2.129

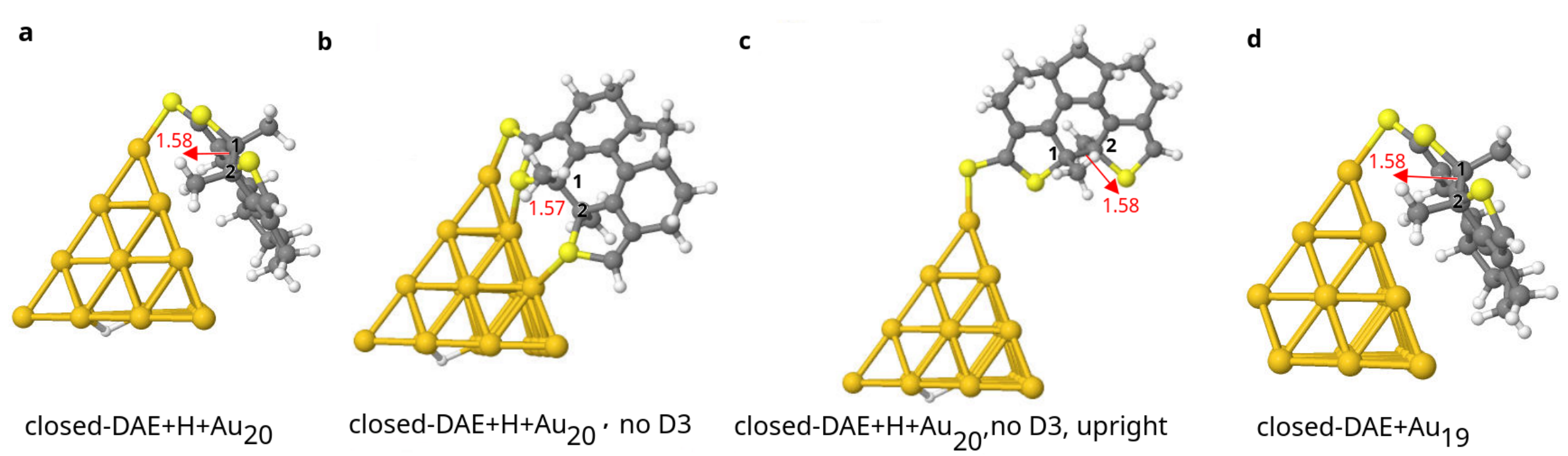

Figure S6: Additional models of closed-form DAE adsorbed on Au_{19} and Au_{20} clusters considered in Table S2. Geometry optimizations were perfromed with B3LYP[+D3(BJ)].

S3 Spin density

To examine the localization of the unpaired electron in the open-shell DAE–Au_n systems, we calculated the spin density for selected systems. Figure S7 shows the spin densities of the open-DAE–Au_{10} and open-DAE–Au_{20} systems. In both cases, the spin density is mainly concentrated on the DAE moiety and the molecule–Au interfacial region, with a smaller contribution delocalized over the Au cluster.

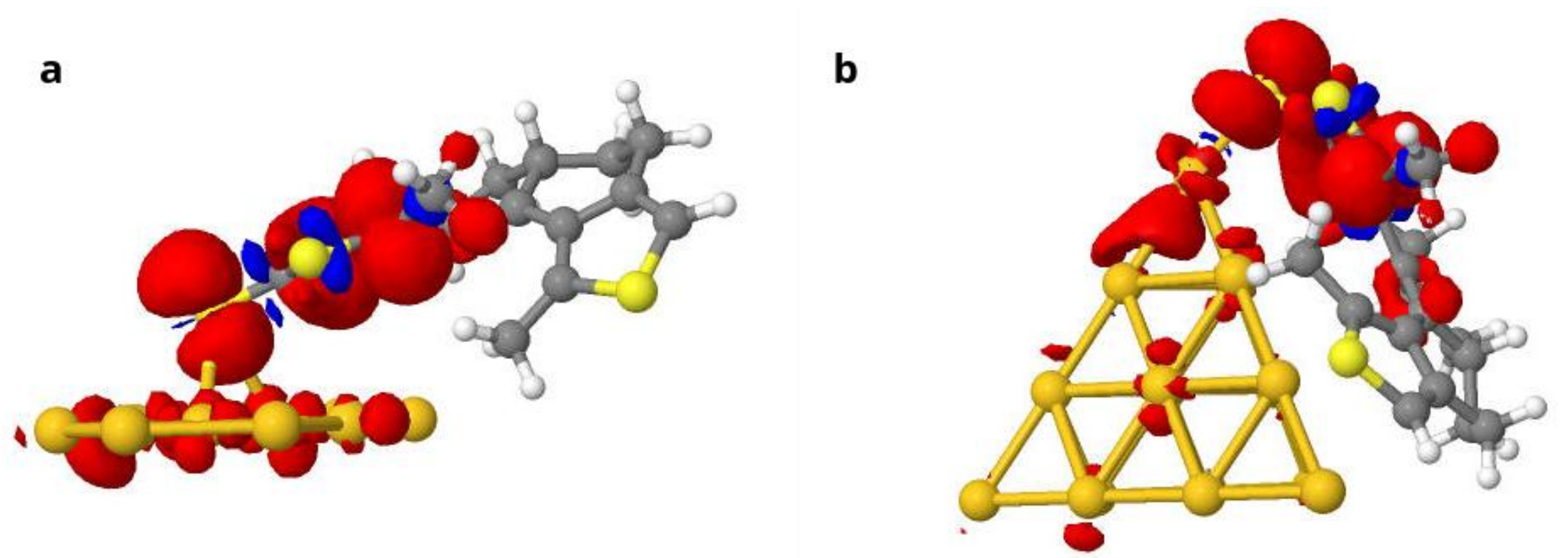

Figure S7: Spin densities of (a) open-DAE–Au_{10} and (b) open-DAE–Au_{20} calculated at the UB3LYP level.
